# A Second Glass Transition Observed in Single-Component Homogeneous Liquids Due to Intramolecular Vitrification

**DOI:** 10.1021/jacs.3c07110

**Published:** 2023-11-18

**Authors:** Ben A. Russell, Mario González-Jiménez, Nikita V. Tukachev, Laure-Anne Hayes, Tajrian Chowdhury, Uroš Javornik, Gregor Mali, Manlio Tassieri, Joy H. Farnaby, Hans M. Senn, Klaas Wynne

**Affiliations:** † School of Chemistry, University of Glasgow, Glasgow G12 8QQ, U.K.; ‡ Slovenian NMR Centre, National Institute of Chemistry, SI-1000 Ljubljana, Slovenia; § Department of Inorganic Chemistry and Technology, National Institute of Chemistry, SI-1001 Ljubljana, Slovenia; ∥ Division of Biomedical Engineering, School of Engineering, University of Glasgow, Glasgow G12 8QQ, U.K.

## Abstract

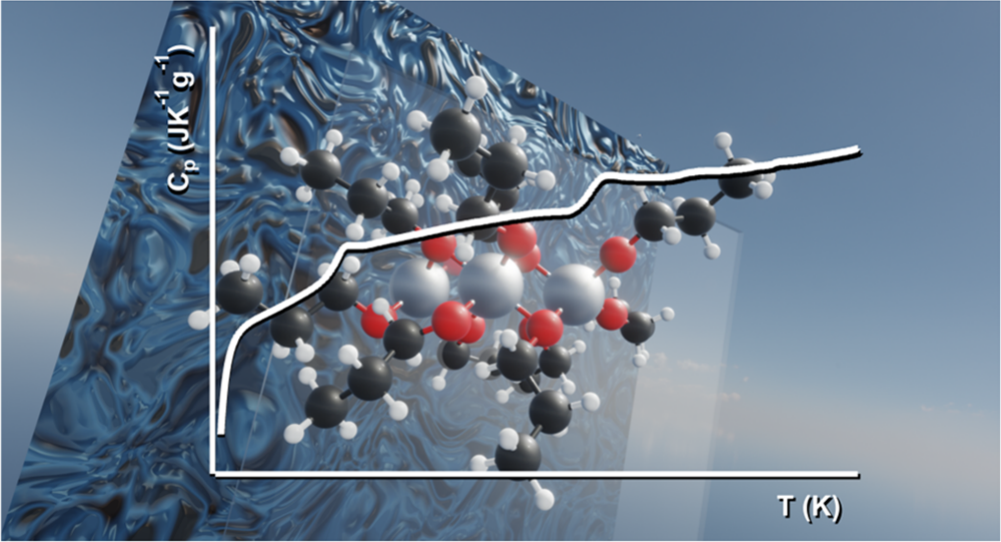

On supercooling a liquid, the viscosity rises rapidly until at the glass transition it vitrifies into an amorphous solid accompanied by a steep drop in the heat capacity. Therefore, a pure homogeneous liquid is not expected to display more than one glass transition. Here we show that a family of single-component homogeneous molecular liquids, titanium tetraalkoxides, exhibit two calorimetric glass transitions of comparable magnitude, one of which is the conventional glass transition associated with dynamic arrest of the bulk liquid properties, while the other is associated with the freezing out of intramolecular degrees of freedom. Such intramolecular vitrification is likely to be found in molecules in which low-frequency terahertz intramolecular motion is coupled to the surrounding liquid. These results imply that intramolecular barrier-crossing processes, typically associated with chemical reactivity, do not necessarily follow the Arrhenius law but may freeze out at a finite temperature.

## Introduction

A glass transition is the kinetic arrest or freezing out of a diffusive degree of freedom. Translational and rotational molecular diffusion rates are inversely proportional to the macroscopic shear viscosity constituting the primary or α relaxation, with small deviations caused by the inhomogeneous nature of the glassy state. The viscosity (η) becomes extremely high (typically defined as η ∼ 10^12^ Pa·s when the primary relaxation time is about 100 s) at a temperature very close to the glass transition temperature (*T*_g_), defined as the temperature at which the heat capacity shows a steep drop in value. At the glass transition, rotational and translational diffusion rates may decouple, with the former remaining inversely proportional to the viscosity and the latter decreasing to a lesser extent.^1^ As both types of molecular diffusion and viscosity are intimately tied up and only decouple at near glasslike viscosities, one expects to observe only one glass transition.

Second glass transitions have been seen in binary glass-forming systems such as methyltetrahydrofuran with tristyrene,^2^ tripropyl phosphate with polystyrene,^3^ and aqueous citric acid,^4^ and even a triple glass transition in the fluoroaluminosilicate Fuji G338 ionomer glass system.^5^ However, in these mixtures, the multiple glass transitions are associated with inhomogeneities and the vitrification of chemically distinct components of the mixture. Similarly, in ionomers consisting of a neutral chain backbone and charged groups, ionic clustering results in inhomogeneities and a broadened and even a double glass transition.^6^ A similar example is the apparent double glass transition observed in some polymers caused by the emergence of partial crystallinity (for example, in polyethylene).^7^ Finally, a double glass transition associated with a liquid–liquid transition has been observed in yttrium–aluminum oxide glasses.^8,9^

Here we show that homogeneous (pure) titanium alkoxide liquids exhibit two calorimetric glass transitions of comparable magnitude: that is, a comparable change in heat capacity. The low-temperature calorimetric transition is a glass transition in the classic sense associated with the freezing out of whole-molecule translational motion (classic primary or α relaxation). We will show that the high-temperature calorimetric glass transition is caused by the freezing out of diffusive intramolecular motions. This effect can be applied to any molecule with intramolecular motions coupled to the surrounding liquid.

## Results

### The Alkoxides

**Figure 1:**
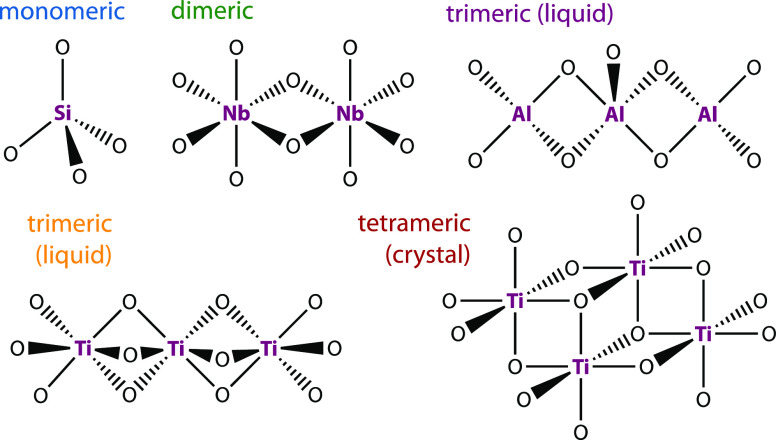
Cartoon structures of four transition-metal alkoxides. Only the oxygen atoms of the alkoxide groups are shown here. Silicon alkoxides have the formula Si(OR)_4_; silicon prefers tetrahedral coordination and is therefore monomeric in the liquid and crystal. Niobium alkoxides have the formula Nb(OR)_5_; niobium prefers octahedral coordination and is therefore dimeric in the liquid and crystal. Titanium alkoxides have the formula Ti(OR)_4_, while titanium prefers octahedral coordination. Due to steric hindrance, these typically form trimers in the liquid. If the alkoxide is short (methoxide and ethoxide), it can crystallize in the tetrameric form. Aluminum alkoxides have the formula Al(OR)_3_; aluminum prefers octahedral coordination, resulting in trimers in the liquid and tetramers in the crystal.

A number of alkoxides based on silicon, niobium, aluminum, and titanium were studied here (see Figure 1). Silicon tetraalkoxides are monomeric in both liquid and crystalline phases. Short chain silicon alkoxides can crystallize, while longer chain ones (e.g., silicon tetrabutoxide) only vitrify into a glass and were recently studied to understand the emergence of the boson peak in molecular glasses.^10^ Pentaalkoxides based on niobium and tantalum are typically six-coordinated, dimeric in both liquid and crystalline phases, and bioctahedral.

Titanium-based tetraalkoxides tend to form oligomeric clusters with (imperfect; see below) octahedral and trigonal bipyramidal symmetry. The titanium alkoxides are tetrameric when a crystal can form (methoxide and ethoxide),^11,12^ monomeric when there is significant steric hindrance (e.g., isopropoxide), and trimeric in the typical liquid.^13^ Titanium alkoxides with propoxide or longer chains do not crystallize at all and are therefore “perfect” glass formers in that sense. All of the alkoxides studied here are liquid at room temperature and do not crystallize during the experiments.

Aluminum isopropoxide is distilled as a trimer but converts to a tetramer and subsequently crystallizes over a period of days to months depending on the storage temperature. It converts back to the trimer upon heating above the crystal melting temperature.^14^ The structure of the trimer is that of a central five-coordinated Al atom with two tetrahedrally coordinated Al atoms bound on either side via two bridging alkoxides each.^15^

### Two Calorimetric Glass Transitions

**Figure 2:**
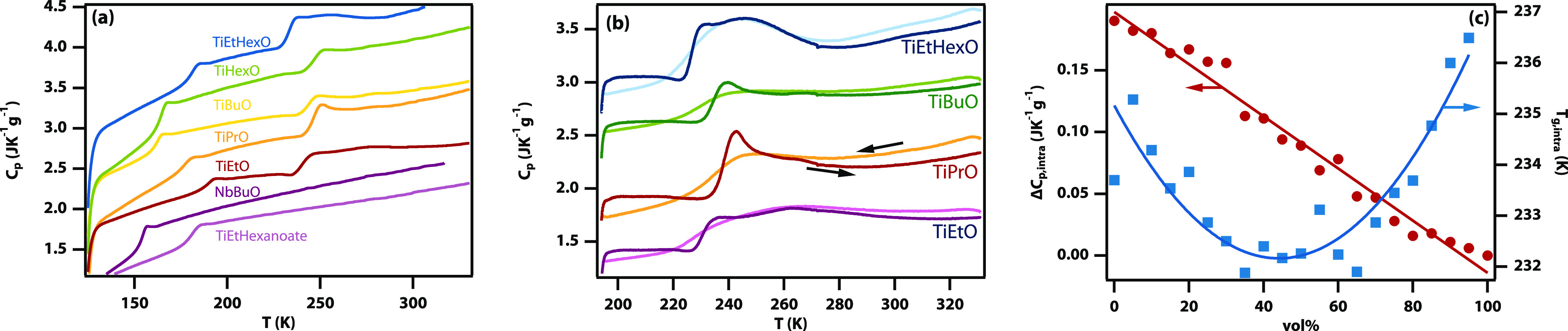
Calorimetry of titanium-based alkoxides shows two calorimetric glass transitions. Heat capacity measurements of titanium ethoxide, propoxide, butoxide, hexoxide, 2-ethylhexyloxide, and 2-ethylhexanoate as well as niobium butoxide. (a) Data obtained using quench cooling with liquid nitrogen to ∼120 K and heating at 20 K/min. See also Table S1. Curves have been shifted vertically for improved visibility. (b) Data obtained using controlled cooling to ∼190 K and heating at 10 K/min. See also Table S2. (c) The magnitude of the change in heat capacity at the second glass transition, Δ*C*_*p-*intra_, as a function of the volume fraction of titanium butoxide on mixing with silicon butoxide.

Monomeric silicon tetrabutoxide has a (single) calorimetric glass transition at *T*_g_ = 120 K,^10^ and other silicon alkoxides also behave as expected. In contrast, trimeric titanium alkoxides show two calorimetric glass transitions (Figure 2) clearly identifiable by step changes in the isobaric heat capacity. The low-temperature glass transition is in all cases at *T*_g_ ≈ 175 K (−100 °C), varying slightly (±15 K) with the alkoxide chain length. The high-temperature glass transition is observed at *T*_g,intra_ ≈ 230 K (−40 °C), again varying slightly (±5 K) with the alkoxide chain length (see Tables S1 and S2). The high-temperature glass transition (which could be studied using controlled cooling and heating at 10 K/min) shows the characteristic smooth transition on cooling. Both transitions show an overshoot on heating, characteristic of a fragile glass former.^16,17^ All the trimeric titanium alkoxides show a bump in the heat capacity ∼15 K above *T*_g,intra_, which in previous work has been associated with a liquid–liquid transition.^18,19^ Trimeric aluminum isopropoxide also exhibits a glass transition at *T*_g_ ≈ 206 K (−67 °C) and a weak high-temperature glass transition at *T*_g,intra_ ≈ 333 K (60 °C) (see Figure S1).

To determine if the multiple calorimetric glass transitions are caused by the non-monomeric nature of the titanium and aluminum alkoxides, niobium ethoxide and butoxide were investigated. While the former tends to crystallize, the latter has a normal (single) glass transition. Similarly, monomeric titanium 2-ethylhexanoate also has a single glass transition. Thus, unusual double glass transitions are observed only in trimeric alkoxides.

The changes in heat capacity at each glass transition are surprisingly large (Δ*C*_*p*-glass_ and Δ*C*_*p-*intra_, see Tables S1 and S2), ranging from 100 to 900 J K^–1^ mol^–1^ at each step. The expected value for Δ*C*_*p*_ in a simple model of a nonspherical incompressible particle is 6*R* ≈ 50 J K^–1^ mol^–1^,^20^ which is indeed observed for many small-molecule glass-forming liquids (for example, 1-propanol Δ*C*_*p*_ ≈ 50 J K^–1^ mol^–1^, methylpentane 70,^21^ 1-butanol 48,^22^ toluene 60, and ethylbenzene 80^23^). The comparatively large values of Δ*C*_*p*_ are observed here only for the trimeric titanium alkoxides. For example, monomeric silicon tetrabutoxide has Δ*C*_*p*_ = 206 J/(K mol) at its (single) glass transition,^10^ similar to monomeric titanium 2-ethylhexanoate (142 J/(K mol)) and dimeric niobium butoxide (261 J/K/mol). A heat-capacity step at the glass transition much larger than 6*R* implies the freezing out of additional intramolecular diffusive motions or alternatively can be related to the thermal expansion coefficient and bulk modulus.^24^ One may speculate that low-frequency (overdamped) vibrations such as alkoxide librations or twisting of the Ti_3_O_12_ core contributes to the Δ*C*_*p*_.

The effect of mixing with monomeric alkoxides, such as silicon butoxide, was investigated (Figure 2(c)). These monomeric alkoxides are chemically stable due to favorable coordination of the silicon atom. In these mixtures, the glass transition temperature at *T*_g,intra_ remains largely unaltered; however, the change in heat capacity, Δ*C*_*p-*intra_, is linearly proportional to the amount of titanium alkoxide present. As these liquids mix well (and therefore do not phase separate), this demonstrates that the second calorimetric glass transition is an intramolecular effect.

### Eliminating Partial Crystallization

Considering that in titanium alkoxides the coordination is different between the liquid and the crystal, it is imperative to determine that the glass transitions are not associated with simple coordination changes or partial crystallization events.

Stretch and bend modes associated with the Ti–O–R motif are observed in the Raman spectrum between 500 and 1500 cm^–1^. In crystalline titanium methoxide, the titanium atom is octahedrally coordinated, resulting in a very simple spectrum in the fingerprint region (see Figure S2). This contrasts with the much more complex spectra of titanium butoxide and 2-ethylhexyloxide. Temperature-dependent Raman spectra of the latter two taken throughout the liquid and glassy range show no major spectral changes on cooling, demonstrating the absence of significant titanium coordination changes (Figures S3 and S4).

Stretch modes associated with CH bonds are observed around 2900 cm^–1^. Subtle changes in the position and amplitudes of these peaks are associated with a transformation from a mixture of trans and gauche orientations at high temperature to predominantly trans alkoxide at low temperature.^25^ This transformation is gradual and does not show steps near the glass transitions.

### Low-Frequency Modes

**Figure 3:**
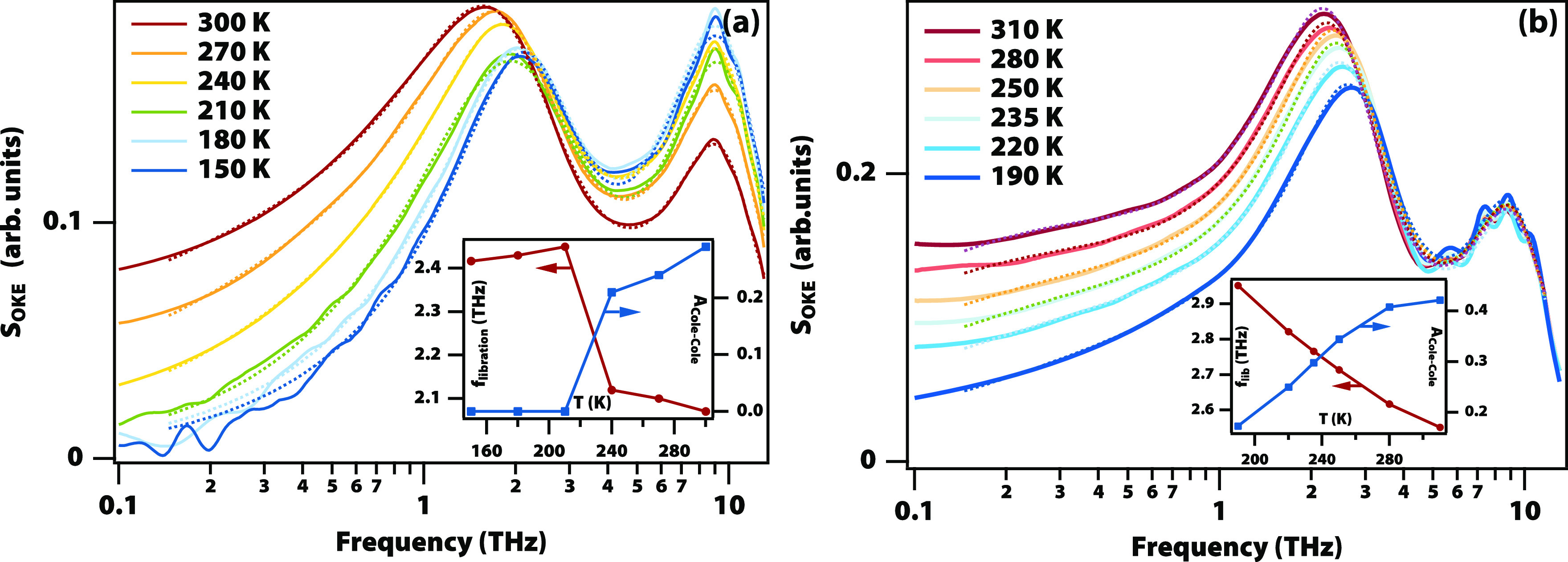
Optical Kerr-effect (OKE) spectra of supercooled and vitrified alkoxide liquids. (a) Data on titanium 2-ethylhexyloxide from 150 to 300 K (solid lines) and fit to a Cole–Cole function representing diffusive modes, a Brownian oscillator (∼1.3 THz) representing alkoxide librations, and a single Brownian oscillator (∼7–8 THz) representing multiple intramolecular vibrational modes. The inset shows the temperature-dependent librational frequency and amplitude of the diffusive mode. (b) Data on niobium ethoxide from 190 to 310 K with similar fits and parameter values in the inset.

The temperature-dependent low-frequency Raman spectra of titanium butoxide and 2-ethylhexyloxide, acquired using femtosecond optical Kerr-effect spectroscopy (see Figure 3(a) and Figure S5(a)),^10,19^ also do not show any phonon bands associated with crystallization. Instead, they show a broad band at 1 to 2 THz and a cluster of narrow vibrational bands at 6–11 THz. The low-frequency band is strongly temperature dependent, showing significant broadening at higher temperatures extending all the way to the lowest accessible frequency of 10 GHz. In the ∼2 THz region, one would expect bands due to the alkoxide intramolecular librations, which are not expected to have a major temperature dependence. The strong temperature dependence implies the presence of a diffusive mode. To model this, the spectra were fitted with two Brownian-oscillator functions—one for the alkoxide librations and one for the vibrations—while the diffusive mode was modeled with a Cole–Cole function (for fit parameters see Tables S3 to S5).^10^ The inset of Figure 3(a) shows the amplitude of the diffusive mode and the frequency of the alkoxide libration, both of which show a large jump at 230 K.

These experiments were repeated on dimeric niobium ethoxide and butoxide (see Figure 3(b) and Figure S5(b)). In this case, there is no jump in the value of any of the parameters but just a gradual narrowing and blue shift on cooling.

### Rheology

The reduction in the heat capacity at the (single) calorimetric glass transition is always associated with a dramatic slowdown of the primary relaxation and hence a dramatic increase in the viscosity.^26^ Given that the titanium alkoxides have two glass transitions, it is of the utmost importance to relate these changes to the rheological behavior.

**Figure 4:**
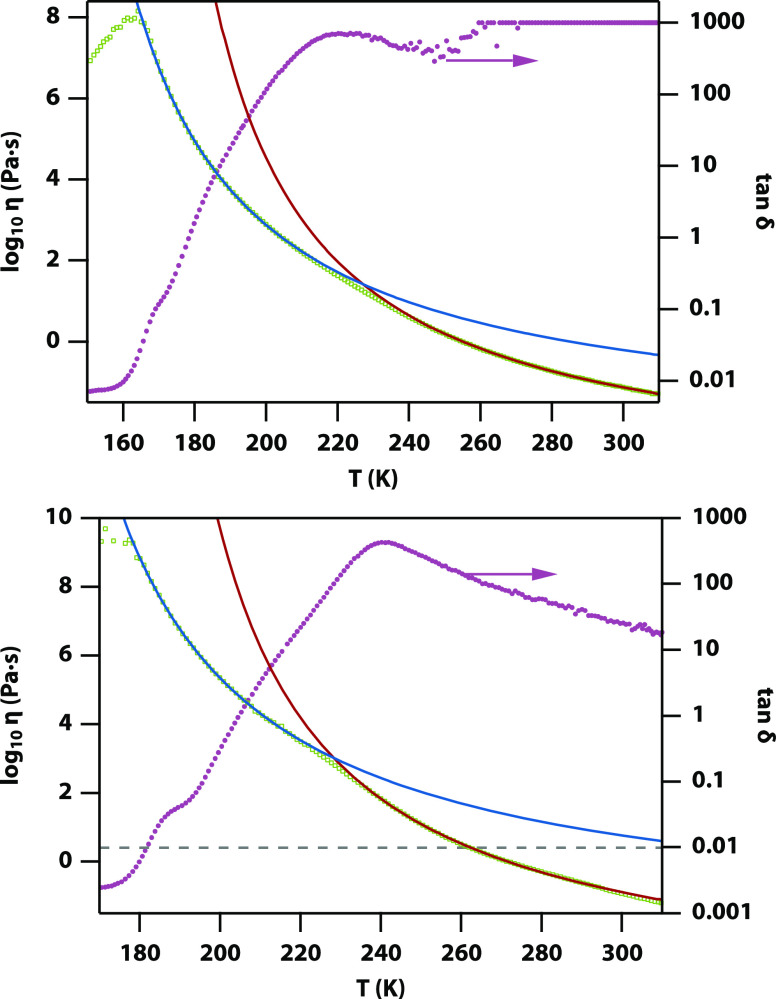
Viscosity measurements of titanium butoxide and titanium 2-ethylhexyloxide. Shear viscosity up to a maximum of ca. 10^10^ Pa·s (green circles) for titanium butoxide (top) and titanium 2-ethylhexyloxide (bottom). The lines are the fits of two separate Vogel–Fulcher–Tammann (VFT) expressions (blue and red lines, parameters in Table S6). The right axis shows the loss tangent.

The shear viscosities of titanium propoxide, butoxide, hexoxide, and 2-ethylhexyloxide as well as niobium butoxide were measured from 313 K (+40 °C) down to a few K above their *T*_g_ (see Figure 4 and Figure S6). The data were fitted with a Vogel–Fulcher–Tammann (VFT) expression, η(*T*) = η_0_ exp(*D*/(*T* – *T*_0_)), where *T*_0_ is the temperature of apparent divergence of the viscosity.^26^ However, none of the viscosities for trimeric alkoxides can be fully modeled by a single VFT function, as all show a clear switch in behavior around 230 K. High-quality fits could be obtained by fitting the low-temperature range (*T*_g_ + 10 to 210 K) and high-temperature range (240 to 300 K) separately (see Table S6 for fit parameters). In all cases, *D* > *T*_0_, consistent with these liquids being moderately fragile glass formers.^27^ The *T*_0_ parameters are in all cases ∼10–60 K below the respective calorimetric *T*_g_, i.e., for both the low (∼175 K) and the high (∼230 K) glass transitions, as expected for glass formers with moderate fragility. In contrast to the trimeric alkoxides, the measured shear viscosity of dimeric niobium butoxide and monomeric titanium 2-ethylhexanoate and silicon butoxide^10^ could be fit well with a single VFT expression.

Temperature-dependent storage and loss moduli were measured using oscillatory rheology for titanium butoxide and titanium 2-ethylhexyloxide (Figure S7). The dynamic viscosity calculated from these is consistent with the shear viscosity. The loss tangent shows a drop from values consistent with a viscous liquid at high temperature to values consistent with an ultraviscous liquid at low temperature with a wide viscoelastic range.

### Isomerization Dynamics and Self-Diffusion

Earlier studies of transition-metal alkoxides have reported “rapid” exchange of terminal and bridging alkoxides.^28^ To establish the temperature-dependent rate of isomerization through ligand exchange, ^13^C magic-angle-spinning (MAS) solid-state NMR was carried out on titanium ethoxide (Figure S8) and titanium 2-ethylhexyloxide (Figure S9). As the former gave the cleanest spectra, we concentrate on these.

The ^13^C MAS NMR spectrum of titanium ethoxide shows lines at ∼70 ppm due to the CH_2_ group next to the oxygen atom and lines at ∼20 ppm due to the terminal CH_3_ group. At low temperature, both are split into multiple lines due to the presence of multiple isomers. At higher temperature these coalesce into single lines due to fast exchange on the NMR time scale.

This coalescence was modeled with a Bloch–McConnell exchange model with the temperature-dependent ligand exchange described by an Eyring equation with as the only free parameter the activation energy (see Supplementary Note 1). This describes the data well for an activation energy of 52.3 kJ/mol for titanium ethoxide and an activation energy of 46 ± 2 kJ/mol for titanium 2-ethylhexyloxide. However, the data can also be modeled with a Vogel–Fulcher–Tammann rate equation with a divergence temperature of 230 K and fragility parameter *D* = 200. The signal-to-noise ratio of a ^13^C NMR experiment is insufficient to distinguish between these two scenarios.

### Structure and Dynamics of the Trimers

Calorimetry, optical Kerr-effect, and rheology data show that the double glass transition, and its ancillary effects, only occurs in trimeric titanium alkoxides but not in dimeric or monomeric equivalents. This suggests an additional quality that is present only in these trimers.

**Figure 5:**
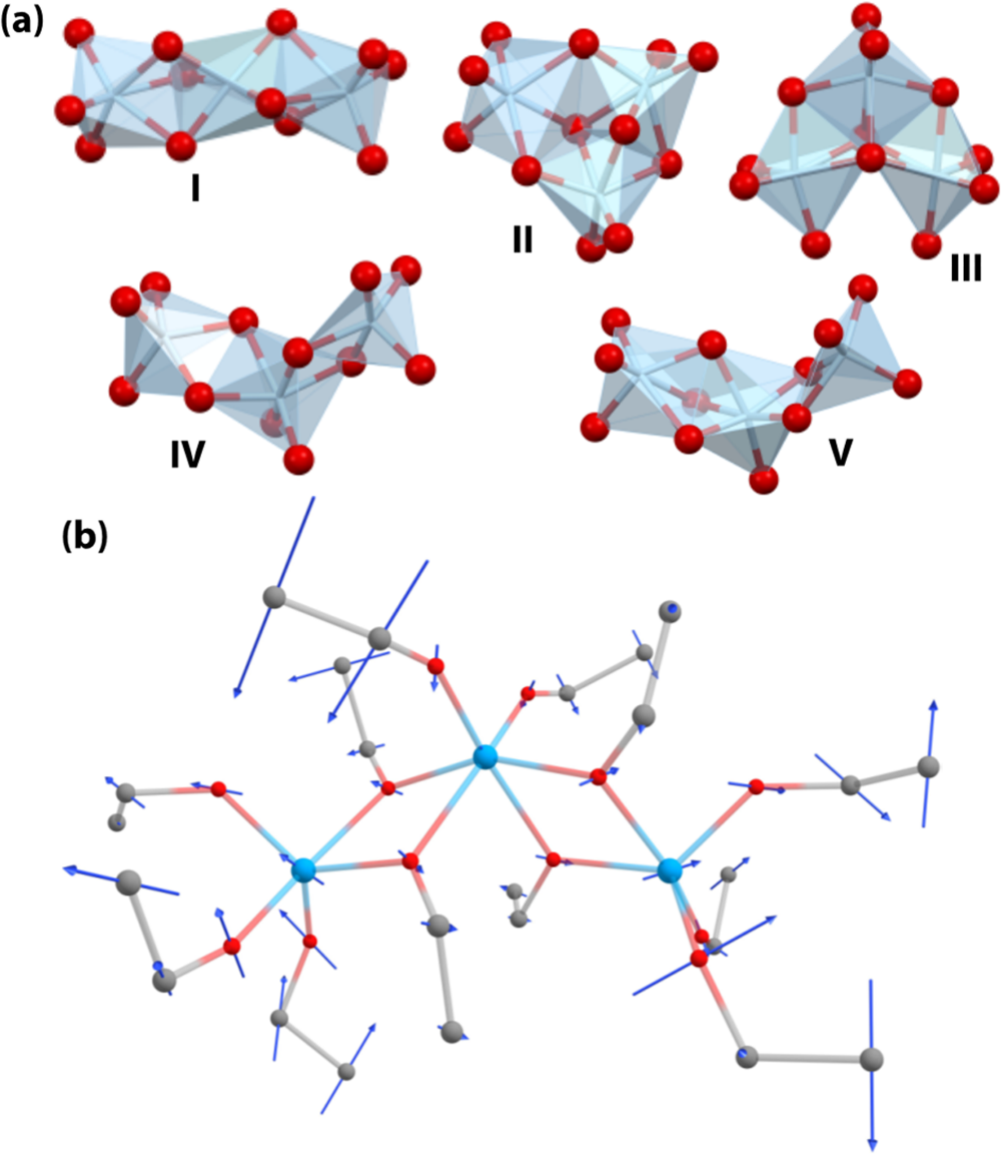
Structure and dynamics of the trimeric alkoxides. (a) Molecular models of the titanium cores of trimeric titanium alkoxides. The five clusters shown have similar energies with the lowest-energy isomer dependent on the alkoxide chain length. Only the titanium (gray) and oxygen atoms (red) are shown. (b) Titanium-core bending modes are strongly coupled to the liquid. Normal-mode calculations show that most vibrational modes between ca. 80 and 150 cm^–1^ (2.5–5 THz) involve Ti_3_-core bending and twisting motions with significant displacement of the terminal carbons of alkoxide side chains causing strong coupling to the surrounding liquid. Shown here is mode 31 (89 cm^–1^/3 THz) in isomer IV as a typical example of this effect.

Quantum chemistry calculations were carried out to establish which trimeric isomers were most likely to be present in the liquid (Supplementary Note 2). Out of a large range of trial structures, five stable isomers were found (Figure 5(a)). For isomers I–III, the coordination number of each titanium atom is six, while the other structures have one or two titanium atoms with a coordination of five. None of the TiO_6_ and TiO_5_ coordination geometries are perfectly octahedral nor trigonal-bipyramidal or square-pyramidal, respectively, but they are notably distorted. Isomers I, IV, and V are essentially linear, whereas II and III are notably bent. As can be seen in Table S7, the energy differences between the isomers range from 0.8 to 32 kJ/mol, and the most stable isomer is either II or III depending on the length of the alkoxide used.

The barriers for isomerization between the five isomers were calculated (for a single titanium ethoxide trimer, see Supplementary Note 3). Unsurprisingly, the barriers for isomerization between linear isomers are low (33–68 kJ/mol), and those for isomerization between bent isomers are also low (22–25 kJ/mol). Barriers for isomerization from bent to linear are higher (74–135 kJ/mol). The predicted time scale for isomerization at room temperature is 1–10 ns between like isomers, which excludes isomerization as a source of the diffusive motion observed experimentally on a time scale of ca. 1 ps.

Vibrational normal-mode analysis was carried out for each of the five isomers. The lowest frequency modes (≤70 cm^–1^) are mostly librational in character, with the Ti_3_O_12_ core librating as a unit. Relatively high frequency modes (≥170 cm^–1^) have significant TiO_5_/TiO_6_ (depending on coordination) stretch character with very little displacement of the alkoxide side chains, explaining why the Raman peaks at 6–11 THz are relatively sharp. Intermediate frequency modes (80–150 cm^–1^ or 2.5–5 THz) involve twisting and bending of the Ti_3_O_12_ core accompanied by very large displacement of alkoxide side chains (see Figure 5(b)). Coupling to the surrounding liquid will cause all of these modes to be damped, with low-frequency large-amplitude modes more likely to be overdamped (rate of damping greater than the mode frequency). Such overdamped modes will then be diffusive in nature; that is, they undergo stochastic motions rather than deterministic vibrations.

### Discussion and Conclusions

Here we have shown that titanium alkoxide liquids exhibit two calorimetric glass transitions with the transition temperatures weakly dependent on the length of the alkoxide chain (2 to 8 carbon atoms). In mixtures of titanium butoxide (*T*_g,intra_ = 234 K) with monomeric and unreactive silicon butoxide (*T*_g_ = 120 K), the second high-temperature glass transition does not change significantly as expected for a normal (intermolecular) glass transition,^29^ while the change in heat capacity scales linearly with the titanium butoxide fraction. This demonstrates the intramolecular character of the second glass transition.

Temperature-dependent Raman spectroscopy confirms that the transitions are not related to any changes in the coordination of the titanium atoms, ruling out partial crystallization. Rheology shows that on cooling from room temperature, the shear viscosity increases in a VFT-like fashion down to *T*_g,intra_ and then continues to rise in a different VFT-like fashion down to *T*_g_. The relatively low viscosity at *T*_g,intra_ (as compared to that at *T*_g_) for the trimeric liquids rules out a decoupling of translational and rotational diffusion as the cause for the second calorimetric glass transition. Although double VFT behavior has been observed previously,^30^ here it is seen in the trimeric alkoxide liquids but not in monomeric (e.g., silicon)^10^ or dimeric (niobium) alkoxides. The latter also does not exhibit the double calorimetric glass transition. This demonstrates that the unusual behavior is not due to just the alkoxide side chains but intrinsic to the trimeric nature of the titanium alkoxides. Given the similarities in mass and size of the dimeric and trimeric molecules, it is highly unlikely that rotational diffusion will freeze out only in the trimeric liquids. It also shows that the low-temperature glass transition at *T*_g_ is the “normal” glass transition at which macroscopic transport ceases.

Unlike monomers and dimers, trimers can bend. Quantum chemistry calculations show that the trimeric titanium alkoxides have twisting and bending modes of the Ti_3_O_12_ core in the 2.5–5 THz range, which are associated with large displacement of the alkoxide side chains. Exactly in this frequency range, optical Kerr-effect experiments find a band that is diffusive at high temperatures and that freezes out on cooling below *T*_g,intra_. We therefore propose that the large displacement of the side chains causes a coupling to the surrounding liquid whose viscosity controls the damping of the diffusive Ti_3_O_12_-core twisting and bending modes.

The standard assumption for intramolecular processes is that any barrier-crossing process follows the Arrhenius law *k* ∼ exp(−*E*_b_/*k*_B_*T*). Here we have shown that this is not always the case, as an intramolecular diffusive process associated with the Ti_3_O_12_-core modes is shown to freeze out at a finite temperature, implying that it follows a VFT-type law, *k* ∼ exp(−*D*/(*T* – *T*_v_), where *T*_v_ is the intramolecular vitrification temperature.

There are numerous reports of calorimetric anomalies that have been associated with liquid–liquid transitions in molecular liquids.^31^ We surmise that some of these, for example, those involving large flexible molecules,^18,32,33^ may find their origin in intramolecular vitrification. Additionally a number of glass-forming metal–organic framework (MOF) liquids containing bulky ligands, such as certain zeolithic imidazolate frameworks (ZIFs)^34,35^ and coordination polymers,^36,37^ have been shown to exhibit calorimetric anomalies at temperatures above the glass transition that may well be related to intramolecular vitrification. The general principle of intramolecular vitrification is the coupling of low-frequency intramolecular modes with large-amplitude motions of parts of the molecule in contact with the surrounding liquid, which is itself vitrifying. This suggests that the effect will be observed much more widely and has a general bearing on material and chemical properties.

## Data Availability

The data that support the findings of this study are available in Enlighten: Research Data Repository (University of Glasgow) with the identifier: 10.5525/gla.researchdata.1525.

## Supporting Information Available

Methods, supplementary data and fit parameter tables, supplementary data figures (calorimetry, Raman spectra, rheology, ^13^C ssNMR, quantum chemistry calculations), supplementary notes (PDF)
