# Supplementary material for: A Second Glass Transition Observed in Single-Component Homogeneous Liquids Due to Intramolecular Vitrification
Source: J Am Chem Soc. 2023 Nov 18;145(48):26061–7. doi: 10.1021/jacs.3c07110 (PMC10704603; doi:10.1021/jacs.3c07110)
Supplement: Supplementary file 1 — ja3c07110_si_001.pdf [file ja3c07110_si_001.pdf]

Supplementary information – A second glass transition observed in single-component homogeneous liquids due to intramolecular vitrification

Ben A. Russell,[1] Mario González-Jiménez,[1] Nikita V. Tukachev,[1] Laure-Anne Hayes,[1] Tajrian Chowdhury,[1] Uroš Javornik,[2] Gregor Mali,[3] Manlio Tassieri,[4] Joy H. Farnaby,[1] Hans M. Senn,[1] Klaas Wynne[1]*

[1]School of Chemistry, University of Glasgow, Glasgow G12 8QQ, UK
[2]Slovenian NMR Centre, National Institute of Chemistry, SI-1000 Ljubljana, Slovenia
[3]Department of Inorganic Chemistry and Technology, National Institute of Chemistry, SI-1001 Ljubljana, Slovenia
[4]Division of Biomedical Engineering, School of Engineering, University of Glasgow, Glasgow G12 8QQ, UK

METHODS

Sample preparation. Liquid titanium(IV) ethoxide (≥97%), propoxide (98%), butoxide (≥97%), 2-ethylhexyloxide (95%), and tetrabutyl orthosilicate (97%), were purchased from Sigma-Aldrich while niobium n-butoxide (99%) and titanium(IV) 2-ethylhexanoate (97%) were purchased from Alfa Aesar and were used without further purification. Titanium hexoxide was synthesized as described in Supplementary note 4.

The alkoxides used here react slowly with water from the air: in seconds in the case of titanium ethoxide to hours in the case of monomeric and dimeric species. This reaction forms a film on the surface of the samples thereby sealing the sample. However, prolonged exposure does cause the formation of oxo-bridged polymeric molecules and the gradual disappearance of the multiple glass-transition effects described here. Therefore, the samples were stored and handled in a glovebox with a dry air atmosphere (dew point < -40°C).

Differential scanning calorimetry. Differential scanning calorimetry (DSC) measurements were carried out with a TA Instruments DSC 2500 differential scanning calorimeter equipped with either a RCS90 cooling system allowing cooling to -90°C or a Quench Cooling Accessory allowing cooling from +40°C to about -165°C with liquid nitrogen in circa 16 minutes. Controlled cooling and heating was carried out at a rate of 10 K/min unless stated otherwise. Samples were prepared and DSC pans sealed in the dry-air glove box.

Raman. Confocal Raman microscopy experiments were performed using a Horiba LabRAM HR confocal microscope system. The excitation source was a linearly polarized 28-mW frequency-doubled DPSS laser operating at 532 nm. Temperature was controlled to ±0.1 K using a Linkam THMS600 microscope stage. The samples were prepared in the dry-air glove box and dispensed into a quartz crucible (18 mm diameter, 3 mm depth) then sealed with an 18 mm diameter cover slip and silicon grease.

OKE experimental details. A laser oscillator (Coherent Micra) produced ~ 10 nJ pulses at a repetition rate of 82 MHz and with 800 nm nominal wavelength providing 20 fs temporal pulse width in the sample, broadening to 25 fs when using a cryostat. The OKE data were recorded in a standard time-domain pump-probe configuration and Fourier transformed to obtain the frequency-domain reduced depolarized Raman spectrum as described previously.[1] The data were analyzed through curve fitting as described previously.[2]

Rheology. Rheology measurements were carried out on an Anton Paar MCR 702e rheometer equipped with a CTD 600 MDR convection temperature device. The temperature-dependent shear viscosity was measured by using a 50-mm cone-plate geometry (cone angle = 1°, gap = 0.101 mm) or a 50-mm parallel-plate geometry (gap = 1 mm) using a shear rate of 0.3 rad/s allowing the viscosity to be measured down a few K above T_g. The temperature-dependent storage ($G'(\omega)$) and loss moduli ($G''(\omega)$) were measured by using a 50-mm parallel-plate geometry and oscillation frequencies of either 1 or 10 Hz. These measurements were used to calculate the complex viscosity, $\eta^* = G(\omega)/i\omega$, and hence the dynamic viscosity $\eta' = \mathrm{Re}\ \eta^*$ in addition the loss tangent $\tan\delta = G''(\omega)/\ G'(\omega)$. The temperature was lowered from 30°C to -160°C at a rate of 1°C/min and the data recorded every minute.

As the alkoxide liquids react slowly with water from the air, a special procedure was used for sample loading. A home-made plastic and rubber cup was placed on the lower measuring plate forming a good seal. The cup was filled with xenon gas, which is heavier than air and therefore keeps the ambient air away from the lower measuring plate. A sample in a micropipette is prepared in the glovebox, transferred to the rheometer, and put on the xenon-covered lower measuring plate. A cone or parallel plate is then lowered through the gas onto the sample. The cup

can then be lifted and the CTD 600 MDR convection temperature device closed, which then flushes dry air through the sample chamber.

MAS NMR. Temperature-dependent ^{13}C magic-angle spinning (MAS) NMR spectra were recorded on a 400 MHz Bruker Avance Neo spectrometer equipped with a 4-mm Bruker CPMAS probe. The investigated liquids were closed into Kel-F inserts before being put into 4 mm zirconia rotors. These samples were prepared in an argon-filled (BOC, 99.998%) glove box (MBRAUN LABstar, $O_2 \leq 5$ ppm, $H_2O \leq 0.5$ ppm). In the MAS-NMR experiments, the samples were spun with a frequency of 10 kHz. A 90° pulse of 3.8 µs was used for the excitation of carbon nuclei, and proton decoupling was employed during signal acquisition. The number of scans was 256 using a repetition delay of 3s and an acquisition to 540 ms to acquire 32k points. The processed data were apodised with 2.5 Hz LB exponential function and baseline corrected with a polynomial. The ^{13}C shifts are reported relative to the position of the ^{13}C signal of tetramethylsilane (TMS).

Isomer stability and normal mode calculations. All calculations were performed using the ORCA 5.0 quantum chemistry program.[3] A preliminary conformational search to find low-energy TiO skeletons for titanium alkoxides trimers was carried out using the semiempirical GFN2-xTB method. Geometry optimizations and vibrational spectral calculations were carried out with the PBE exchange-correlation functional with the Grimme dispersion correction (D3)[6] with Becke-Johnson damping[7] combined with def2-SVP basis set.[8] PBE is a nonempirical GGE functional applicable to a broad range of systems from simple organic molecules to inorganic solids and metals, while the def2-SVP basis set is known to provide a good tradeoff between accuracy and cost. To estimate errors arising due to basis set incompleteness, calculations with triple-zeta (def2-TZVP, def2-TZVPP) basis sets were also performed. To validate the energy ordering of different titanium alkoxide trimers, PBE0-D3(BJ)/def2-TZVPP calculations (geometry optimizations starting with converged PBE-D3(BJ)/def2-SVP structures) were conducted. Solvent effects were accounted for throughout by using the CPCM polarizable continuum model[9] with a dielectric constant of $\varepsilon_{static} = 1.89$ (hexane). For all DFT calculations, the RI approximation was employed.

It is known that titanium ethoxide and butoxide form trimers in neat liquid and solution (except at high dilution).[10–12] In order to identify low-energy trimers, various Ti_3O_{12} cores were cut from crystal structures of rutile and anatase TiO_2 polymorphs, saturated with hydrogens, and optimized with the GFN2-xTB method, yielding four different isomers. Next, the hydrogens were substituted by methyl, ethyl, or butyl, and DFT optimizations were performed. Additional Ti_3O_{12} cores were obtained from $Ti(OR)_4$ crystal structures from the Cambridge Structural Database. Their optimization as $Ti_3(OH)_{12}$ yielded one more stable isomer, thus leading to five different minima on the PES (see Figure 5 of the main text, Supplementary note 2, and Figure S10). All geometry optimizations were performed without symmetry constraints. Ti_3O_{12} cores were found to be close to S_6 (**I**), C_2 (**III**, **IV**) and C_s (**II**, **V**) symmetry. Harmonic analyses of these structures showed that all Hessian eigenvalues are positive. The optimized structures **I**–**V** were compared to those proposed in the literature based on general considerations[12] or theoretical studies.[13] Our structures **I** and **III** correspond to I and II of Refs. [11,13]; structure **IV** is known from crystal trimers. However, it appears that structures **II** and **V** have not previously been considered. Structures III and IV of Ref. [12] are not stable and rearrange to one of the five stable isomers identified herein during geometry optimization.

Although a full conformational analysis of the alkyl chains in solution for all studied alkoxides was not carried out, it was decided to investigate conformer energy distributions for Ti ethoxide trimers (Figure S11). For this purpose, 50 ps GFN2-XTB MD runs were conducted for each of the skeletons (NVT ensemble, Berendsen thermostat, 300 K, log-fermi potential to confine a trimer in a spherical cavity to prevent possible dissociation). Ten snapshots were then extracted from resulting trajectories and optimized with PBE0-D3(BJ)/def2-TZVP, CPCM (hexane) resulting in 50 DFT-optimized structures in total. For methoxides and butoxides, the optimized alkyl conformations are to some extent arbitrary; butyl chains have the all-trans "zig-zag" conformation. Enthalpies and Gibbs energies were estimated within the rigid rotator–harmonic oscillator model.

Normal modes and IR and depolarized Raman spectra were obtained in the double harmonic approximation and PBE0(D3BJ)/def2-SVP, CPCM (hexane); Raman data were converted to OKE spectra using a Bose-Einstein correction as described previously.[2] Spectra were broadened with Lorentzians with a 12 cm^{-1} half-width (see Figure S12 and Figure S13).

SUPPLEMENTARY TABLES

Table S1. *Calorimetric glass transition temperatures and heat capacity changes of both glass transitions. DSC data obtained using quench cooling to ~120 K and heating at 20 K/min. DSC data are in Figure 2.*

	$M_{monomer}$ / g mol^{-1}	$M_{effective}$ / g mol^{-1}	T_g / K	$\Delta C_{p\text{-glass}}$ / JK^{-1}mol^{-1}	$T_{g,intra}$ /K	$\Delta C_{p\text{-intra}}$ / JK^{-1}mol^{-1}	$\Delta C_{p,total}$ / JK^{-1}mol^{-1}
**Titanium ethoxide**	228.15	684.45	189.2	103.4	240.4	180.0	283.4
**Titanium propoxide**	284.22	852.66	176.6	163.7	245.8	289.1	452.8
**Titanium butoxide**	340.32	1020.96	162.1	222.6	243.2	232.8	455.3
**Titanium hexoxide**	442.46	1327.368	164.0	512.4	245.8	351.8	864.2
**Titanium 2-ethylhexyloxide**	564.75	1327.38	179.7	404.9	234.2	648.9	1053.8
**Niobium butoxide**	458.12	1129.5	154.0	260.5	n/a	n/a	n/a
**Titanium 2-ethylhexanoate**	623.75	623.75	182.0	142.2	n/a	n/a	n/a
**Aluminum isopropoxide**	204.25	612.74	-67.0	161.8	60.0	74.1	235.9

Table S2. *Calorimetric glass transition temperatures and heat capacity changes of the high temperature glass transition only. DSC data obtained using controlled cooling to ~190 K and heating at 10 K/min. DSC data are in Figure 2.*

	$M_{monomer}$ / g mol^{-1}	$M_{effective}$ / g mol^{-1}	T_g / K	$\Delta C_{p\text{-glass}}$ / JK^{-1}mol^{-1}	$T_{g,intra}$ /K	$\Delta C_{p\text{-intra}}$ / JK^{-1}mol^{-1}
**Titanium ethoxide**	228.15	684.45	-	-	230.7	222.4
**Titanium propoxide**	284.22	852.66	-	-	237.3	219.1
**Titanium butoxide**	340.32	1020.96	-	-	233.7	327.7
**Titanium 2-ethylhexyloxide**	564.75	1694.25	-	-	226.7	867.5
**Niobium butoxide**	458.12	916.24	-	-	n/a	n/a
**Titanium 2-ethylhexanoate**	623.75	623.75	-	-	n/a	n/a

Table S3. *OKE fit parameters titanium 2-ethylhexyloxide.*

T (K)	150	180	210	240	270	300
**A_1**	0	0	0.145± 0.005	0.212±0.004	0.244±0.007	0.29±0.01
**τ^{-1} (THz)**	0	0	12.5±0.5	11.8±0.4	4.8±0.3	2.0± 0.1
**α**	0	0	0.93±0.01	0.71±0.01	0.60±0.01	0.58±0.01
**A_2**	110±2	130±2	61±4	75±2	93±2	111±3
**γ_2 (THz)**	26.5±0.8	38±2	21±1	24.3±0.7	33±2	88±33
**f_2 (THz)**	1.33±0.02	0.96±0.03	1.32±0.04	1.12±0.02	0.84±0.03	0.3±0.1
**A_3**	516±9	492±6	294±5	261±5	264±8	228±8
**γ_3 (THz)**	85±2	78±2	57.6±0.8	54.9±0.7	60±1	60±1
**f_3 (THz)**	7.18±0.09	7.38±0.06	8.41±0.03	8.27±0.03	8.06±0.04	8.01±0.05

Table S4. *OKE fit parameters niobium ethoxide.*

T (K)	190	220	235	250	280	310
A_1	0.17±0.02	0.25±0.02	0.30±0.03	0.34±0.03	0.41±0.03	0.42±0.02
τ^{-1} (THz)	3.9±0.7	2.2±0.2	1.9±0.1	1.64±0.07	1.32±0.05	1.35±0.04
α	0.72±0.05	0.69±0.05	0.68±0.05	0.67±0.04	0.67±0.04	0.72±0.03
A_2	0.184±0.004	0.198±0.004	0.205±0.004	0.210±0.004	0.210±0.003	0.218±0.003
γ_2 (THz)	2.92±0.07	2.87±0.06	2.85±0.06	2.83±0.06	2.78±0.05	2.73±0.05
f_2 (THz)	2.95±0.02	2.82±0.01	2.77±0.01	2.71±0.01	2.62±0.01	2.55±0.01
A_3	0.149±0.005	0.140±0.004	0.142±0.004	0.140±0.004	0.140±0.004	0.139±0.003
γ_3 (THz)	7.5±0.2	7.3±0.2	7.3±0.2	7.3±0.2	7.4±0.2	7.5±0.2
f_3 (THz)	9.56±0.04	9.61±0.05	9.56±0.05	9.56±0.05	9.60±0.05	9.63±0.05

Table S5. *OKE fit parameters niobium butoxide.*

T (K)	160	190	220	235	250	280	310
A_1	0.13±0.01	0.23±0.02	0.30±0.03	0.35±0.04	0.37±0.04	0.46±0.05	0.49±0.06
τ^{-1} (THz)	6.0±0.42	4.7±0.4	3.0±0.2	2.6±0.2	2.25±0.07	1.82±0.05	1.61±0.07
α	1.04±0.02	0.88±0.03	0.83±0.05	0.81±0.05	0.85±0.05	0.86±0.06	0.88±0.06
A_2	0.185±0.005	0.187±0.006	0.200±0.007	0.203±0.007	0.217±0.007	0.230±0.007	0.230±0.007
γ_2 (THz)	2.80±0.05	2.89±0.06	3.06±0.07	3.11±0.08	3.19±0.07	3.26±0.08	3.33±0.08
f_2 (THz)	2.677±0.006	2.56±0.01	2.48±0.01	2.43±0.01	2.39±0.01	2.30±0.01	2.24±0.01
A_3	0.240±0.002	0.229±0.004	0.226±0.005	0.230±0.006	0.228±0.004	0.222±0.005	0.226±0.004
γ_3 (THz)	9.00±0.08	8.7±0.2	8.8±0.2	8.7±0.2	8.7±0.2	8.9±0.2	9.2±0.2
f_3 (THz)	9.23±0.02	9.16±0.03	9.27±0.03	9.21±0.03	9.29±0.03	9.31±0.03	9.32±0.03

Table S6. *Fits to the temperature-dependent shear viscosity using a Vogel-Fulcher-Tammann function. The function used is* $\eta = \eta_0\, exp(D/(T - T_0))$. *The data below and above 230 K (-40°C) were fitted separately as described in the main text. The fits are shown in Figure 4 and Figure S6.*

	η_0 (low T) / Pa·s	D (low T) / K	T_0 (low T) / K	η_0 (high T) / Pa·s	D (high T) / K	T_0 (high T) / K
**Titanium propoxide**	0.0006±0.0001	1319±18	135.3±0.4	0.013±0.002	330±19	202.9±1.5
**Titanium butoxide**	0.0041±0.0005	857±15	129.2±0.5	0.00025±.00002	819±15	156.3 ± 1.1
**Titanium hexoxide**	0.0082±0.0007	727± 9	134.7±0.3	5.7 10^{-6}±4.2 10^{-5}	1236±1.6 10^{3}	152±68
**Titanium 2-ethylhexyloxide**	0.008±0.003	1062±46	137.9±1.2	8.9 10^{-5}±1.4 10^{-5}	947±30	170.1±1.4
**Niobium butoxide**	0.0014±0.0001	811±13	123.2±0.4	n/a	n/a	n/a
**Titanium 2-ethylhexanoate**				n/a	n/a	n/a

Table S7. *Energies (relative to the most stable isomer) for the isomers I–V of Ti(OR)4 trimers (R = Me, Et, n-Bu) shown in Figure S10 as obtained by PBE0-D3(BJ)/def2-TZVPP,CPCM(n-hexane).*

kJ/mol	I	II	III	IV	V
			**Me**		
**ΔE**	19.6	15.0	0.0	12.1	30.5
**ΔH_{298}**	19.2	18.8	0.0	13.4	32.2
**ΔG_{298}**	17.6	28.4	0.0	6.3	32.6
			**Et***		
**ΔE**	18.8	23.0	0.0	2.5	32.6
			**n-Bu***		
**ΔE**	30.5	0.0	0.8	5.0	20.9

* PBE0-D3(BJ)/def2-TZVPP values for ethoxides and butoxides correspond to structures resulted from geometry optimization; hessians for these structures were not computed.

Table S8. *Energies (in kJ/mol, relative to the lowest-energy isomer at each level) for titanium ethoxide trimers estimated in chloroform (ALPB solvation model for GFN2-xTB and CPCM for DFT and ab initio).*

	I	II	III	IV	V
**GFN2-xTB**	40.1	2.9	0.0	5.9	40.5
**DFT**	46.9	20.1	11.7	0.0	57.8
**MP2**	38.8	8.6	0.3	0.0	54.1
**CCSD**	37.7	18.9	13.9	0.0	55.3
**CCSD(T)**	38.3	10.9	7.4	0.0	52.3

Table S9. *Highest barriers (in kJ/mol) on the isomerization pathway estimated by GFN2-xTB method (ALPB, chloroform). Values correspond to transitions from the skeleton indicated in the corresponding column (left) to skeleton in the row (top).*

	I	II	III	IV	V
**I**	-	77.8	73.7	32.9	35.9
**II**	114.8	-	21.5	137.2	132.2
**III**	113.7	24.6	-	113.1	137.5
**IV**	67.2	134.6	107.3	-	68.0
**V**	35.3	94.5	96.7	33.0	-

SUPPLEMENTARY FIGURES – CALORIMETRY

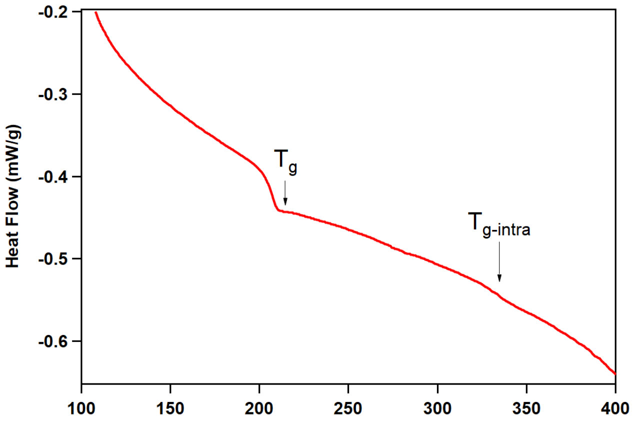

Figure S1. Calorimetry of aluminum isopropoxide shows two calorimetric glass transitions. Data obtained using quench cooling with liquid nitrogen to ~100 K and heating at 10 K/min. Prior to cooling, sample was isothermal at 400 K for 1 hr to fully convert from the tetrameric solid to liquid trimer. Calorimetric glass transitions are observed at $T_g \approx 206$ K (-67.0°C, $\Delta C_{p\text{-}glass} = 162.8$ JK^{-1}mol^{-1}) when supercooled at 10 K/min after holding at 403 K for three hours and a weak second glass transition at $T_{g,intra} \approx 333$ K (60.0°C, $\Delta C_{p\text{-intra}} = 74.1$ JK^{-1}mol^{-1})

SUPPLEMENTARY FIGURES – RAMAN SPECTRA

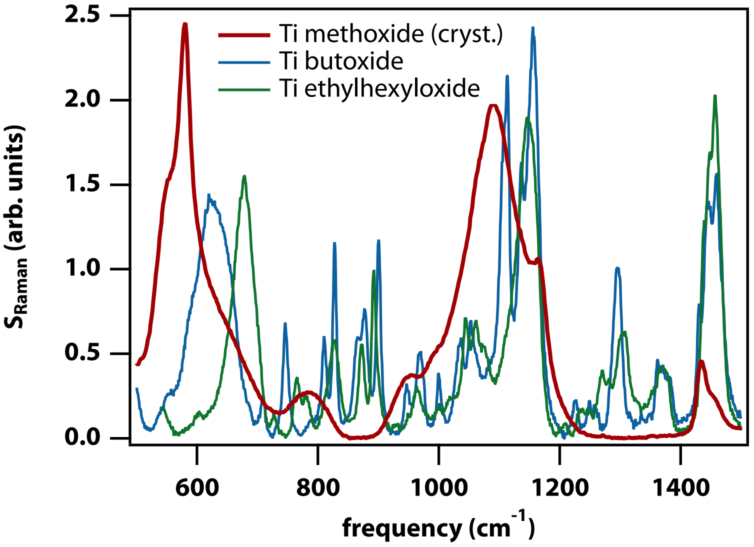

Figure S2. Room temperature Raman spectra of crystalline titanium methoxide and liquid titanium butoxide and 2-ethylhexyloxide in the 500–1,500 cm^{-1} region. The spectrum of titanium methoxide is relatively simple due to the strict octahedral coordination of the titanium atoms in the tetrameric crystalline state. The spectra of titanium butoxide and 2-ethylhexyloxide are much more complicated due to a mixture of coordination numbers in the trimeric liquid.

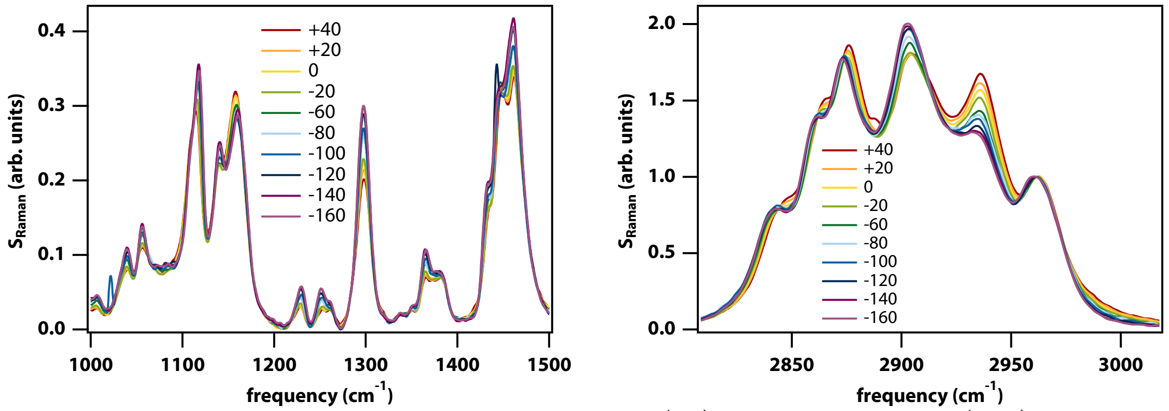

Figure S3. Temperature-dependent Raman spectra of titanium butoxide. (**left**) The fingerprint region and (**right**) the CH-stretch region. The spectra have been normalized at 2961.5 cm^{-1}.

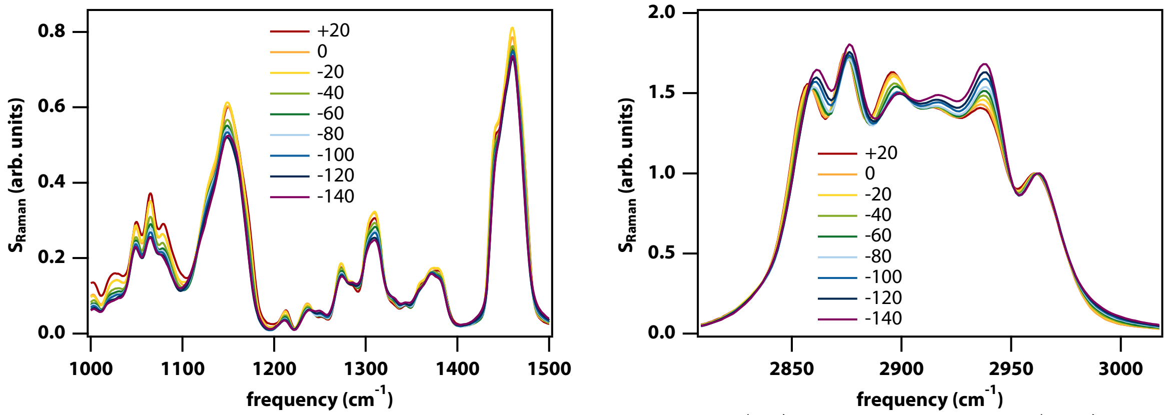

Figure S4. Temperature-dependent Raman spectra of titanium 2-ethylhexyloxide. (**left**) The fingerprint region and (**right**) the CH-stretch region. The spectra have been normalized at 2961.5 cm^{-1}.

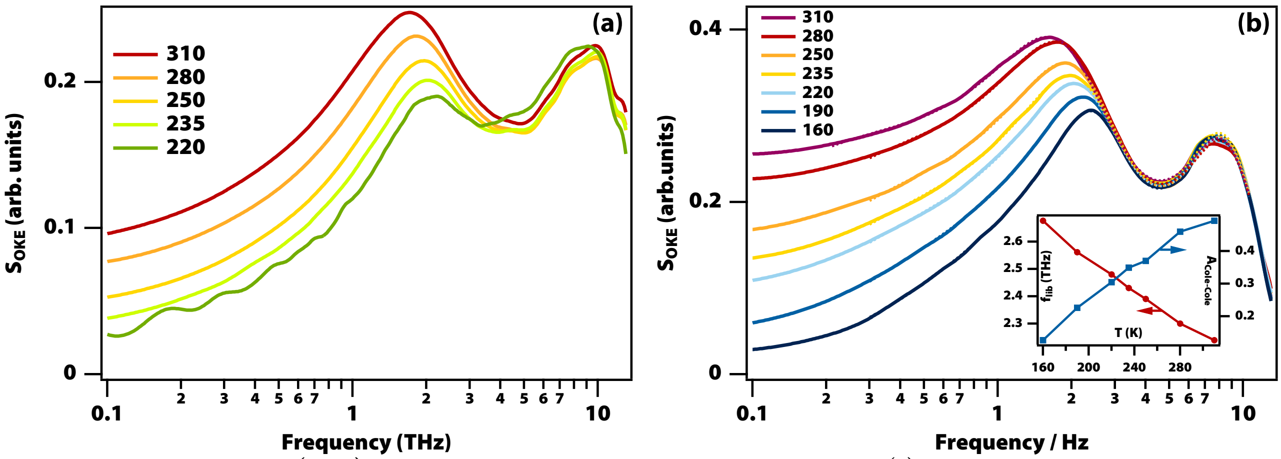

Figure S5. Optical Kerr-effect (OKE) spectra of supercooled and vitrified alkoxide liquids. (**a**) Data on **titanium butoxide** from 220 to 310 K. We were unable to obtain meaningful data below 220 K as the signal-to-noise decreased drastically due to scattering caused by sample shrinkage causing it to peel of the cuvette wall. However, it can be seen that titanium butoxide shows a similar trend as a function of temperature as observed in titanium 2-tehylhexyloxide in Figure 3(a). (**b**) Data on **niobium butoxide** from 160 to 310 K with similar fits as in Figure 3 and parameter values in the inset. The spectral resolution in these data is less than in Figure 3.

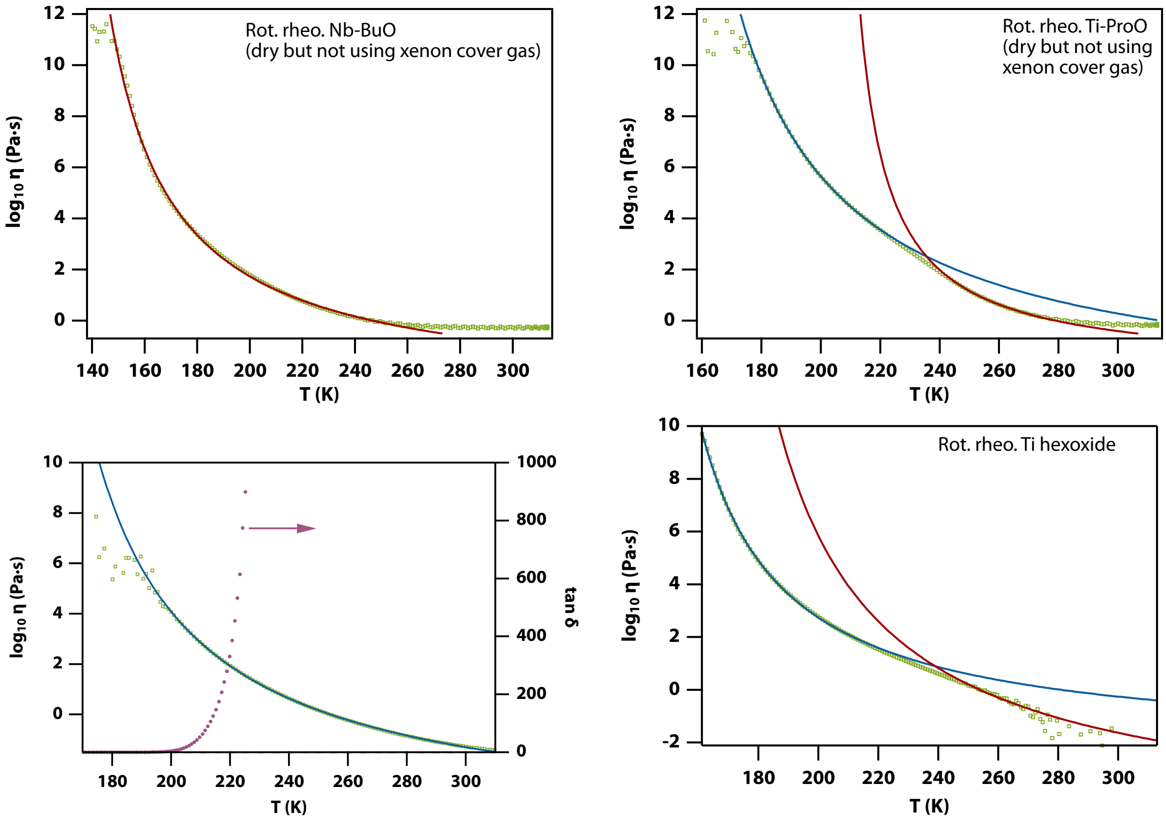

Figure S6. Shear viscosity measurements of a number of transition-metal alkoxides. Shown are data for niobium butoxide, titanium propoxide, titanium 2-ethylhexanoate, and titanium hexoxide.

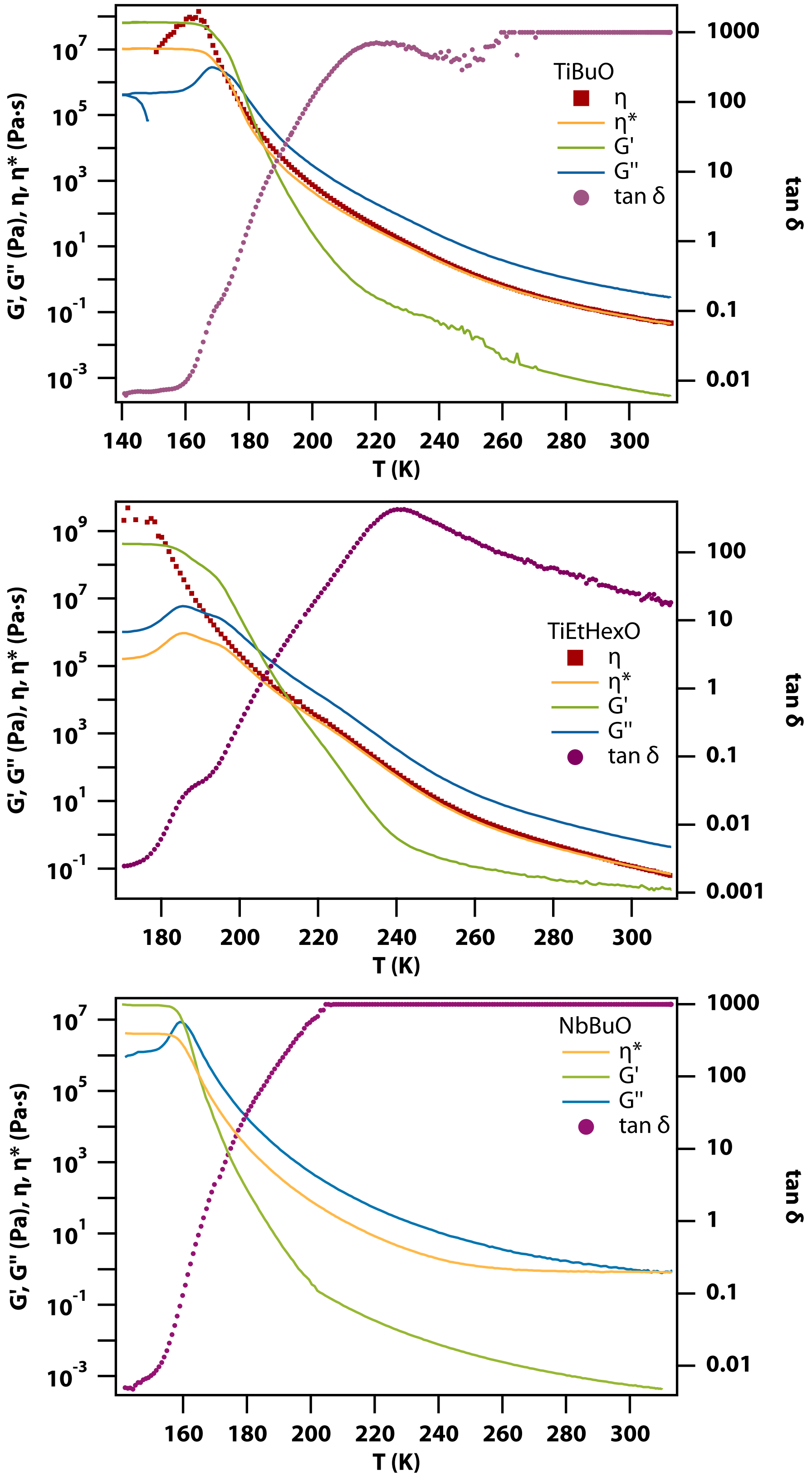

Figure S7. Rheology measurements of the storage and loss moduli of titanium butoxide, 2-ethylhexyloxide, and niobium butoxide. Shown are the measured G' and G'', the calculated loss tangent (tan δ), and the calculated complex viscosity. The later compares well with the measured shear viscosity.

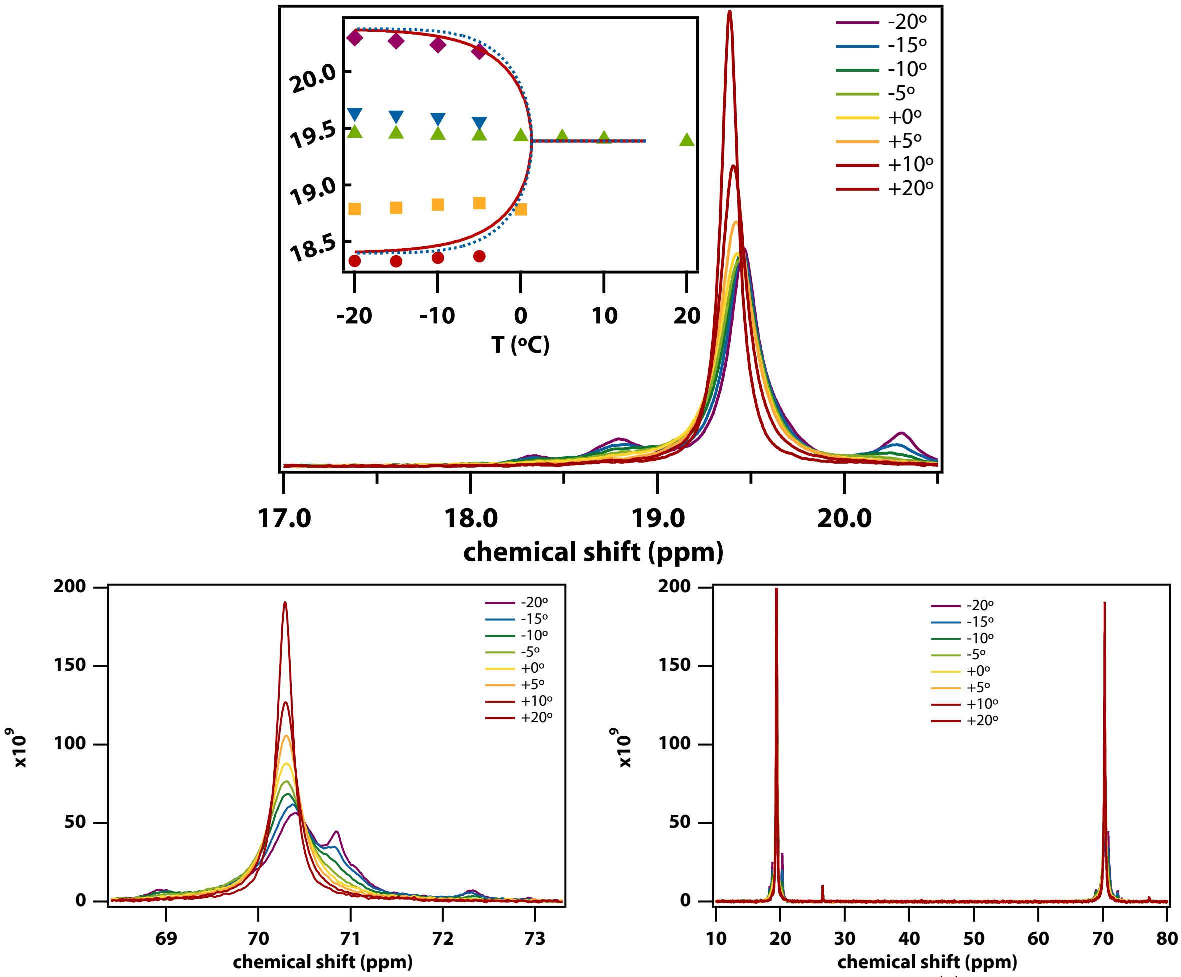

Figure S8. ¹³C MAS NMR spectra of titanium ethoxide. Shown are spectra taken between -20° and 20°C. (**a**) The region of the terminal CH_3 groups. (**a, inset**) Temperature-dependent peak positions and Bloch-McConnell modelling of the line positions using an Eyring equation with an activation energy of 52.3 kJ/mol (red solid line) and using a Vogel-Fulcher-Tammann equation with a divergence temperature of 230 K and *D* = 200 (blue dotted line). (**b**) The lines near 70 ppm are due to the CH_2 group in titanium ethoxide. (**c**) Overview of the spectrum.

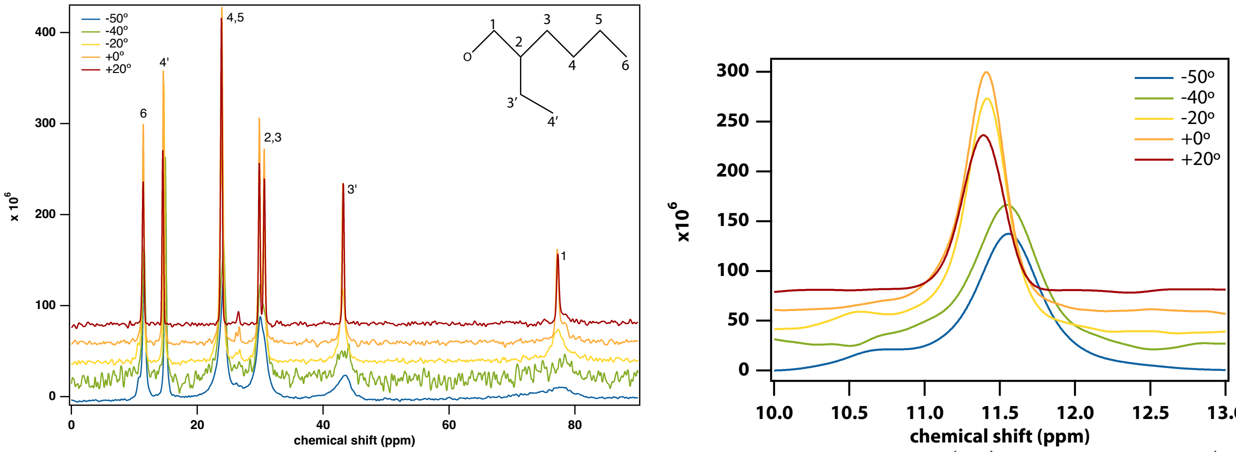

Figure S9. ¹³C MAS NMR spectra of titanium 2-ethylhexyloxide. Temperatures -50, -40, -20, 0, and 20°C. (**left**) Overview of spectrum. (**right**) A shift due motional narrowing occurs between -20° and -40°C consistent with an activation energy of 46±2 kJ/mol.

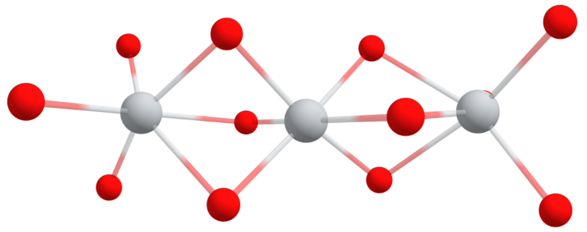

- 3 distorted TiO_6 octahedra connected face-to-face such that the Ti–Ti–Ti angle is close to 180° (2 shared faces)
- 6 μ_2 and 6 terminal oxygens
- S_6 point symmetry group

I

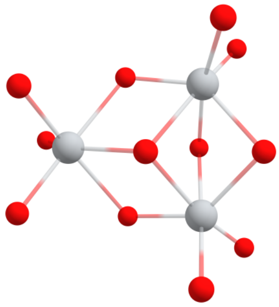

- 2 distorted TiO_6 octahedra connected face-to-face + TiO_6 octahedron sharing 2 edges with each of these two
- 1 μ_3, 4 μ_2 and 7 terminal oxygens
- C_s point symmetry group

II

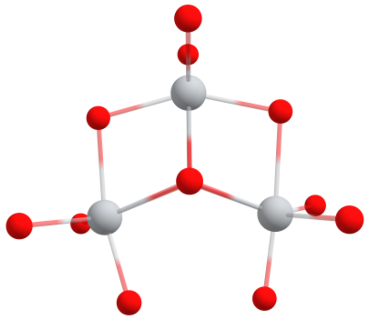

- 3 distorted TiO_6 octahedra connected face-to-face with each other (3 shared faces)
- 2 μ_3, 2 μ_2 and 8 terminal oxygens
- C_2 point symmetry group

III

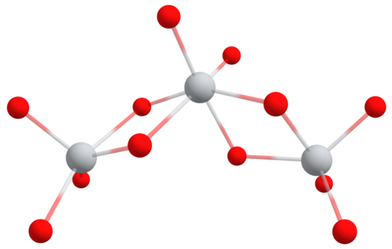

- 1 distorted TiO_6 octahedron sharing 2 edges with 2 square-pyramidal TiO_5
- 4 μ_2 and 8 terminal oxygens
- C_2 point symmetry group

IV

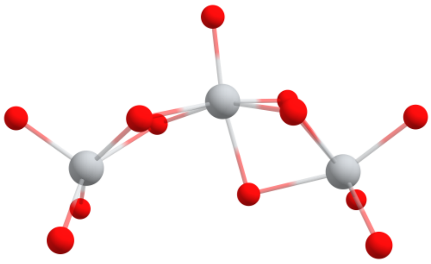

- 2 distorted TiO_6 octahedra connected face-to-face + 1 TiO_5 square pyramid sharing an edge with the “central” TiO_6
- 5 μ_2 and 7 terminal oxygens
- C_s point symmetry group

V

Figure S10. Ti_3O_{12} cores for different isomers of $Ti(OR)_4$ trimers (R = Me, Et, n-Bu).

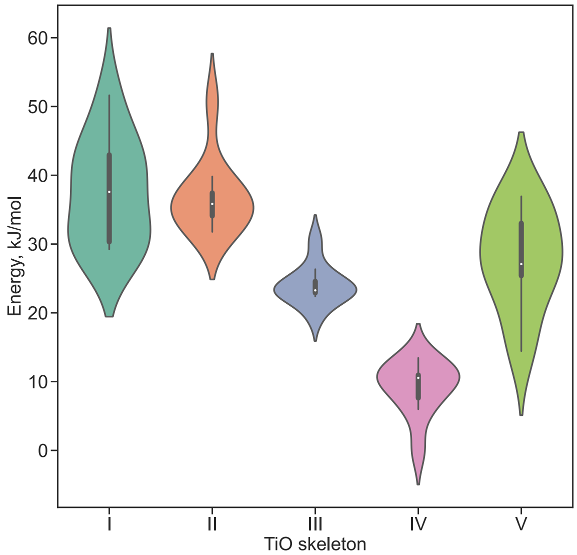

Figure S11. Violin plots for conformer energy distributions for different TiO skeletons of $Ti(OEt)_4$ trimers. Energies estimated by PBE0-D3(BJ)/def2-TZVP, CPCM(hexane)

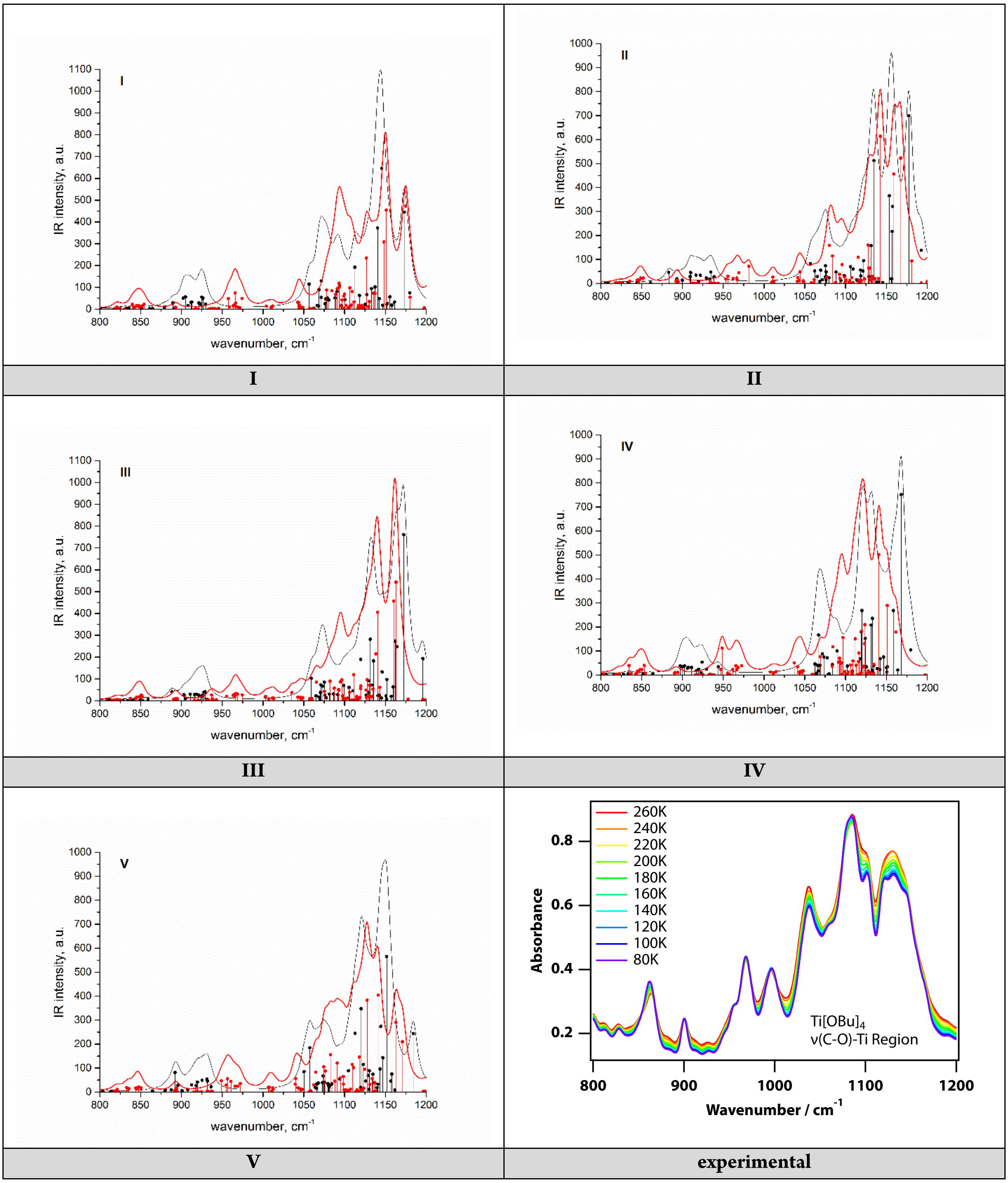

Figure S12. Computed and experimental spectra of titanium butoxide in the mid-IR. This region shows bands due to C-O-Ti stretching/bending modes. Theoretical spectra computed by PBE/def2-SVP for different $Ti(OR)_4$ trimers (R = Et (black), n-Bu (red)).

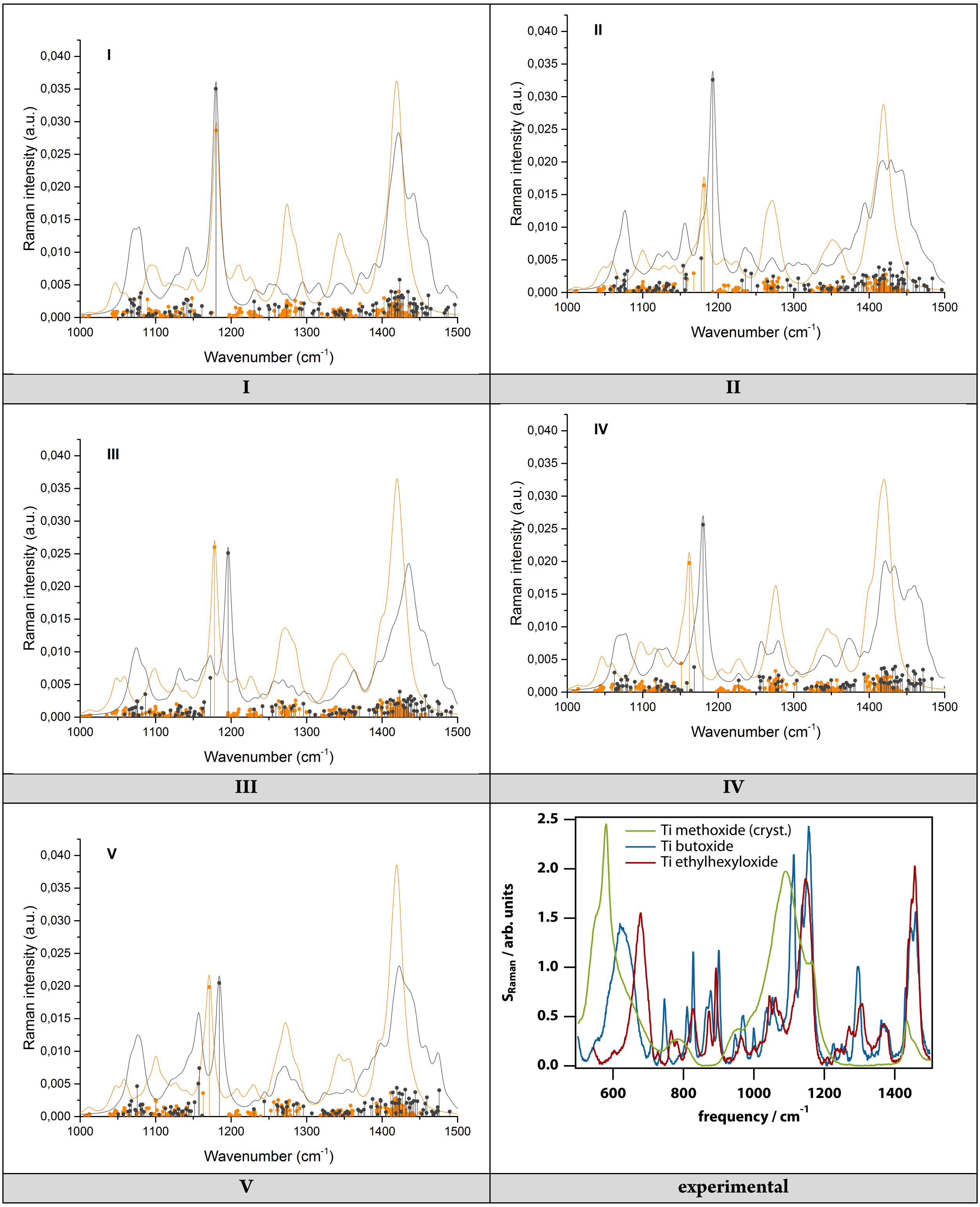

Figure S13. Computed and experimental Raman spectra of titanium alkoxides in 1000-1500 cm⁻¹ region. This region shows bands due to C-O-Ti stretching/bending modes. Theoretical spectra computed by PBE/def2-SVP for different $Ti(OR)_4$ trimers (R = Et (dark grey), n-Bu (orange)).

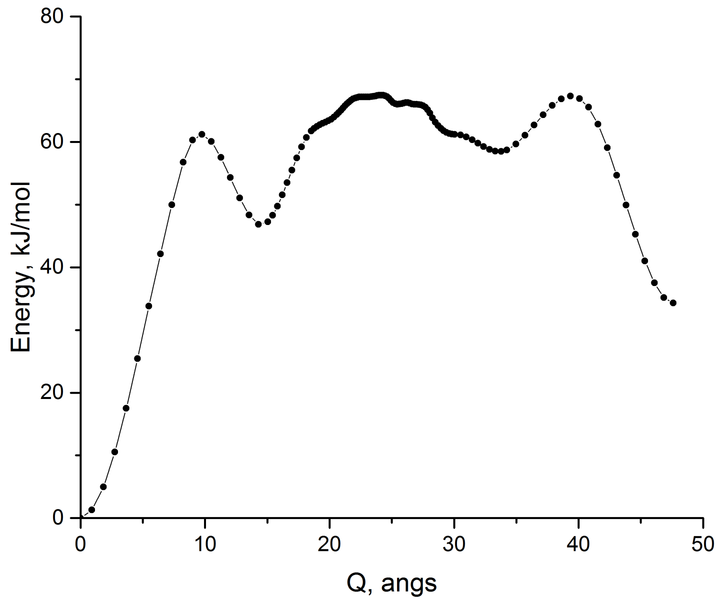

IV-I

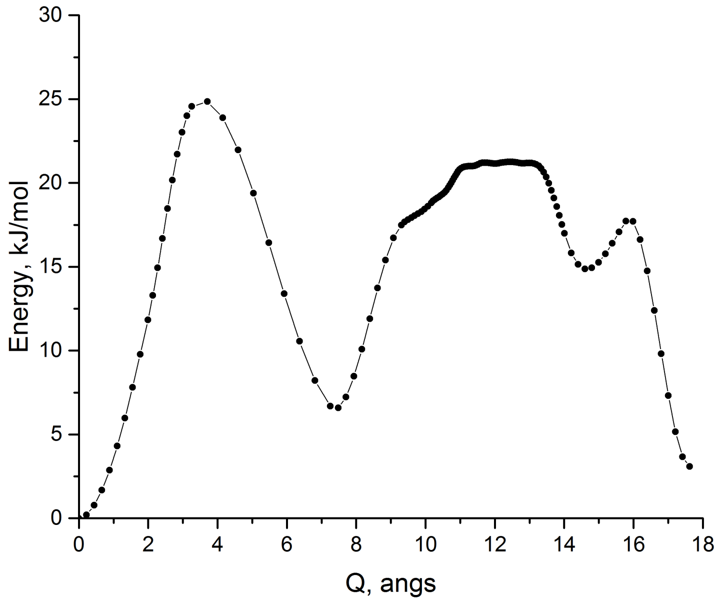

III-II

Figure S14. Estimated (Zoom-NEB-CI) minimal energy paths for IV-I and III-II transitions on GFN2-xTB PES. 116 images were generated during Zoom-NEB-CI procedure for IV-I transition and 125 for III-II transition (not including end points). Intermediate minima correspond to processes involving alkyl chains rotations.

SUPPLEMENTARY NOTES

Supplementary note 1
Bloch-McConnell *vs.* translational diffusion

The Bloch-McConnell equation describes the merging of two NMR spectral lines due to rapid exchange with rate k. The frequencies are given by

$$\omega_{\pm} = \frac{1}{2}(\omega_1 + \omega_2) \pm Re\sqrt{\{\frac{1}{4}(\omega_1 - \omega_2)^2 - k^2\}},$$

where ω_1 and ω_2 are the angular frequencies of the peaks of the unperturbed lines, and $\omega_{\pm}$ are the angular frequencies with the exchange.

It would be reasonable to assume that the rate of ligand exchange is activated and described by an Arrhenius equation. However, the Arrhenius equation has two parameters, which would be difficult to determine over the limited accessible temperature range. Therefore, an Eyring expression

$$k_{Eyring} = \frac{k_B T}{h} \exp\left(-\frac{E_B}{RT}\right)$$

was used where E_B is an activation enthalpy to be determined through fitting to the data and it is assumed that the change in entropy can be ignored. The temperature at which two lines merge is a very strong function of the barrier energy as can be seen in Figure S15.

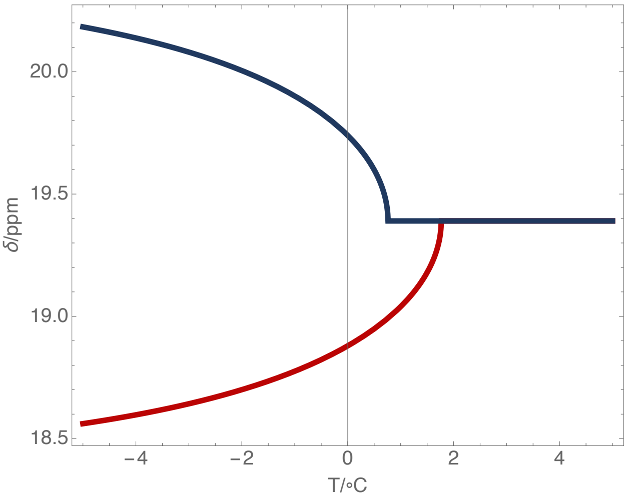

Figure S15. Calculated upper and lower NMR peak shift for barrier heights of 52.2 kJ/mol (upper) and 52.4 kJ/mol (lower).

Alternatively, a Vogel-Fulcher-Tammann rate expression can be used

$$k_{VFT} = \text{A} \exp\left(-\frac{D}{T - T_0}\right)$$

The divergence temperature was set to T_0 = 230 K, while A was set to force a collapse of the NMR multiplet at +1°C, leaving the fragility parameter D as the only adjustable parameter. A good match with the NMR data was found for D = 200.

A translational relaxation rate is calculated using the Stokes-Einstein equation for the translational diffusion coefficient

$$D_T = \frac{k_B T}{6\pi\eta R},$$

where η is the viscosity and R the molecular radius, which is calculated from the molecular mass m and liquid density ρ as

$$R = \sqrt[3]{\frac{3}{4\pi}\frac{m}{\rho N_A}},$$

where N_A is Avogadro's number. The time for diffusion over one molecular diameter is given by

$$\tau_T = \frac{(2R)^2}{6D_T},$$

which gives a diffusion rate of

$$k_T = \frac{k_B T}{4\pi R^3 \eta}.$$

Supplementary note 2
Calculation of stable structures of titanium alkoxides

A search for stable conformers was carried out as described in the Methods section. Among the predicted structures (see Figure S10), for I-III the coordination number for all titanium atoms is 6, and for other two, either one (V) or two (IV) titanium atoms are coordinated by 5 oxygens. It is important to stress, however, that in all these structures, the TiO_6 and TiO_5 cores are notably distorted: they do not form perfect octahedra nor trigonal bipyramids. One can imagine structures I–III and V formed from a dimer of TiO_6 octahedra stacked face to face, adding a third one in such a way as to form a linear chain (I) or to have two adjacent edges and one common vertex (II) or, finally, to have three shared faces and two common vertices (III); V is formed by adding a trigonal bipyramid sharing one edge. Structure IV is comprised of distorted TiO_6 octahedron with two trigonal TiO_5 bipyramids attached.

The structures predicted here are at variance with the conclusions of Ref. [11] based on XANES/EXAFS data, that the stable structure in the liquid phase has all three titanium atoms with 5-fold (D3h) coordination. We believe that this discrepancy can be attributed to the aforementioned distortions from ideal TiO_6 octahedral coordination characteristic for all structures predicted here; for example, for the terminal TiO_6 "octahedron" in structure I, the TiO distances range from 1.78 to 2.35 Å. As a result, the coordination is probably more difficult to establish experimentally for these clusters compared to "simpler" complexes. It is easy to see that all these structures possess somewhat different local environments for oxygens (see Fig. 9) and therefore NMR ^{17}O data could be helpful in identifying the correct conformer(s) in solution.

Relative energies, enthalpies and Gibbs free energies of the predicted structures are presented in Table S7. Generally, structure V is among the highest in energy and is therefore least likely to be observed in solution. For titanium methoxide, it seems safe to assume that conformer III is dominant in solution. For titanium ethoxide, energy differences between structures III and IV are relatively small, and the correct ordering is beyond the accuracy of theory. It is likely that both these structures are present in comparable amount in solution. In previous work[13] it

was proposed on the basis of B3LYP/6-31G* calculations that trimers exist either in structure I or III (in our notations). While we obtain qualitatively similar trends (structure III is lower in energy, but the gap is greatly reduced if the entropy is accounted for), based on our calculations the energy difference is still notable enough to expect that structure I could be excluded from consideration as well as V. For titanium butoxide, our calculations predict that structure II is formally the lowest in energy, and PBE0/def2-TZVPP calculations show that III and IV (to lesser extent) should be taken into account as well. Thus, the prediction here is that—unlike in the crystal phase (where it exists)—titanium ethoxide and butoxide occur as a mixture of conformers II (only for n-Bu), III, and IV. The closeness of the energies of these notably different structures combined with low barriers to conformational transitions between them might be one of the reasons behind the large fragility of the supercooled liquid and the difficulty in obtaining crystals of these compounds.

Because of the differences in local environments for the oxygen atoms, it might be possible to use vibrational spectroscopy to determine what species exist in solution. It can be shown in the case of TiEtO that enlarging the basis set (from def2-SVP to def2-TZVP) leads to a red shift and a reduction in the (absolute) intensity of the vibrational spectra. However, the relative intensities are similar and therefore def2-SVP should provide a reasonable picture for qualitative conclusions at a reduced computational cost. However, the IR spectra of the different structures of TiBuO in the CO-stretch region (from approximately 950 to 1250 cm^{-1}, see Figure S12) are not sufficiently different to make a definitive assignment as to which structure is most prevalent in the liquid phase.

Supplementary note 3
Isomerization energies of titanium alkoxides

In order to assess the viability for titanium alkoxides to interconvert between the isomers **I**–**V**, we estimated the corresponding energy barriers. First, 5 structures exemplifying 5 different TiO skeletons were selected (from the previously studied 50 conformers, see above) and optimized on semiempirical GFN2-xTB level, and then with PBE-D3(BJ)/def2-SVP with CPCM(chloroform) solvation model. To assess DFT accuracy, DLPNO-MP2/cc-pVTZ and DLPNO-CCSD(T) single point calculations in cc-pVTZ basis set were carried out on top of DFT geometries. From the 50 structures found (see above), the structure with the lowest energy was chosen. Then, simple deformations (*e.g.* 'linearizing' Ti-Ti-Ti angle to get to the structure **I**) were applied followed by GFN2-xTB geometry optimizations. This approach was utilized to get 5 isomers that were located relatively close to each other PES-wise. Since we aim to find saddle points characteristic to transitions between isomers, we would like to receive a shorter reaction path with fewer barriers. Because of that, 5 structures studied in the present supplementary section do not exactly coincide with the lowest-energy conformers for each isomer that we found before.

In the main part, we used CPCM (hexane) as a solvation model to estimate, say, conformer energy differences. Provided the information on dielectric constant for $Ti(OEt)_4$ is not available, it might have been interesting to see how PES would change after a relatively small increase in dielectric constant. Hence, these 5 structures were further optimized with PBE-D3(BJ)/def2-SVP with CPCM(chloroform) solvation model. Upon visual inspection of the resulted structures, it was confirmed that trimers skeletons were moderately sensitive to the increase in polarity of the medium geometry-wise and more so energy-wise. It should be noted though that the qualitative considerations for energy ordering of isomers are not affected by the choice of a solvent – at least within PCM solvation model. Thus, to assess DFT accuracy this time, DLPNO-MP2/cc-pVTZ and DLPNO-CCSD(T) single point calculations in cc-pVTZ basis set were carried out on top of DFT geometries.

Corresponding relative energies are presented in Table S8. Having in mind the accuracy of the methodologies used, one can see that these methods agree reasonably well predicting structures **II**, **III** and **IV** to be lower in energy (all within 5 kcal/mol window irrespective of the method used), while structures **I** and **V** are notably higher. This qualitative result allows us to use the xTB method in search of highest energy saddle point on minimal energy paths corresponding to all 10 possible transitions.

In order to obtain an estimate of the reaction path connecting any two isomers and the highest barrier on that path, we performed climbing image NEB (NEB-CI) calculations. We used the Zoom-NEB-CI method[14] along with the FIRE optimizer[15] and the DNEB approximation.[16,17] To ensure tighter convergence to the actual saddle point, the maximum force acting on the CI was converged to 2×10^{-4} Hartree/Bohr and the RMS force to 10^{-4} Hartree/Bohr. Resulting MEPs usually have a few intermediate minima due to alkyl chains being obviously involved in the isomerization process; the number of images created in Zoom variant usually exceed 100 (*e.g.*, Figure S14). From the viewpoint of TiO skeleton rearrangements, all corresponding processes are relatively simplistic—for instance, IV–I involves the increase of Ti–Ti–Ti angle with subsequent formation of two new TiO bonds, or for II-III 2 TiO bonds for 2 distorted octahedra with shared faces break an instead new 2 TiO bonds are formed so that previous octahedra share faces with the third one (not just edges).
— however, due to presence of radicals with flexible dihedrals, prediction of the "exact" path becomes too complicated even on semiempirical PES (see). Values of highest energy saddle points obtained from these calculations (that could be taken as "effective" activation energies although MEPs are quite complex, and all isomerizations are not elementary acts) are listed in Table S9.

Analyzing the data, we note that the barriers to conformational transitions between forms **II** and **III;** and forms (**I**, **IV**, **V**) are notably lower than those for other (say, **I - II**) transitions.

Supplementary note 4
Synthesis of Titanium hexoxide

General Experimental Considerations

All air-sensitive manipulations were carried out in an MBraun glovebox (O_2 < 0.1 ppm and H_2O < 0.5–1.5 ppm) or by using standard Schlenk techniques under N_2. All glassware was dried at 130–135°C overnight prior to use. An Innovative Technology Inc. Pure Solv 400-5-MD solvent purification system (activated alumina columns) was used to obtain anhydrous toluene. Anhydrous toluene was degassed, sparged with N_2, and stored in ampoules over activated 3.0-Å molecular sieves (25-35% weight

by volume) under N_2. Dryness was confirmed by using a sodium benzophenone ketyl solution after 24-48 hours. Deuterated benzene was dried by directly transferring from sealed glass ampoules onto activated 3.0 Å molecular sieves and stored in ampoules in a N_2 atmosphere glovebox. Dryness was confirmed by ^{1}H NMR after 48 hours. Deuterated chloroform (99.8% *d*-atom) was purchased from Cambridge Isotope Laboratories, Inc. and stored under ambient conditions and used without further purification. The following starting materials were purchased from Alfa Aesar: 1-hexanol (99% liquid) and [Ti(NMe$_2$)$_4$] (99.9% metal basis). 1-hexanol was degassed by freeze-pump-thaw degassing cycles (×5) and stored in a pencil ampoule over activated 3.0-Å molecular sieves in a N_2 atmosphere glovebox. The yellow liquid [Ti(NMe$_2$)$_4$] was stored in a pencil ampoule in a N_2 atmosphere glovebox and used without further purification.

Physical Methods

^{1}H NMR data were recorded on an AVIII 400 MHz spectrometer operating at a frequency of 400.1 MHz and the NMR data were referenced internally to the appropriate residual proteo-solvent and reported relative to tetramethylsilane ($\delta\delta$ = 0 ppm). All spectra were recorded at a constant temperature of 25°C (298 K). Coupling constants (*J*) are reported in hertz (Hz). Standard abbreviations for multiplicity were used as follows: m = multiplet, t = triplet, d = doublet, s = singlet. For broad intensities, abbreviated as br, the full width at half-maximum intensity (FWHM) is provided in Hz. ATR-IR spectra were collected in air at ambient temperature using ThermoFisher Scientific Nicolet Summit LITE FTIR Spectrometer (containing a $LiTaO_3$ detector) equipped with Everest ATR. Abbreviations for the intensity of stretching frequencies were used as follows: s = strong, m = medium, w = weak. Elemental analysis was performed by Orla McCullough at the London Metropolitan University, using a Flash 2000 Organic Elemental Analyzer, Thermo Scientific analyzer. The samples for the measurements were prepared using V_2O_5 (to ensure complete combustion of all complexes) in tin capsules inside an inert argon glovebox atmosphere.

Synthesis of [Ti(OCH$_2$CH$_2$CH$_2$CH$_2$CH$_2$CH$_3$)$_4$] by protonolysis of [Ti(NMe$_2$)$_4$] with 4 eq of 1-hexanol

In the glovebox, a 20 mL scintillation vial was charged with a stirrer bar, and then the deep yellow liquid [Ti(NMe$_2$)$_4$] (262 □L, 249.8 mg, 1.11 mmol, 1.0 eq) was added by a micropipette. In a separate vial, a toluene (0.5 mL) solution of the colorless alcohol 1-hexanol (556 □L, 452.8 mg, 4.43 mmol, 4.0 eq) was prepared by weighing out the alcohol using a micropipette. To the [Ti(NMe$_2$)$_4$] liquid, the toluene solution of 1-hexanol was added, by pipette dropwise over two minutes, with stirring at ambient temperature. Dense fumes of the amine HNMe$_2$ were observed, and the immediate formation of a dark orange color was noted, which was stirred at ambient temperature for an hour, after which fuming had subsided and the solution became yellow. Toluene (0.5 mL) was removed *in vacuo*, to yield a dense yellow oil, which was dried *in vacuo* (10^{-2} mbar, 3.5 h) yielding [Ti(OCH$_2$CH$_2$CH$_2$CH$_2$CH$_2$CH$_3$)$_4$] (442.8 mg, 0.978 mmol, 88%) as a yellow oil. ^{1}H NMR (d_6-benzene): δ0.97 (12H, t, $^3J_{H\text{-}H}$ = 7.0 Hz, C**H**$_3$) 1.44 (16H, m, TiO(CH$_2$)$_3$(C**H**$_2$)$_2$CH$_3$) 1.55 (8H, approx. quintet, $^3J_{H\text{-}H}$ = 7.5 Hz, TiO(CH$_2$)$_2$C**H**$_2$) 1.93 (8H, br s, FWHM = 23.3 Hz, TiOCH$_2$C**H**$_2$) 4.68 (8H, br s, FWHM = 22.5 Hz, TiOC**H**$_2$) ppm. ^{1}H NMR (*d*-chloroform): δ 0.89 (12H, t, $^3J_{H\text{-}H}$ = 6.7 Hz, C**H**$_3$) 1.30 (24H, m, TiO(CH$_2$)$_2$(C**H**$_2$)$_3$CH$_3$) 1.60 (8H, approx. quintet, $^3J_{H\text{-}H}$ = 6.9 Hz, TiOCH$_2$C**H**$_2$) 4.24 (8H, br s, FWHM = 36.6 Hz, TiOC**H**$_2$) ppm. Anal. Calcd. for $C_{24}H_{52}O_4Ti$: C, 63.70%; H, 11.58%; N, 00.00%. Found: C, 57.04-57.38%; H, 10.09-10.61%; N, 0.21-0.28%. Note: A small % of N is observed in the product owing to minute HNMe$_2$ impurities, resulting in slight discrepancies with the calculated values. Lower CHN values than calculated may be the result of incomplete combustion. However, the observed CH ratio $C_{24}H_{52}$ approximately matches the calculated. IR (ATR): 3070-3596 (w, υ_{OH}),* 2953 (s, $\upsilon_{sp^3\text{-}CH}$), 2923 (s, $\upsilon_{sp^3\text{-}CH}$), 2857 (s, $\upsilon_{sp^3\text{-}CH}$), 1468 (m), 1382 (m), 1124 (s), 1089 (s), 1057 (s), 1023 (s), 921 (m), 724 (s), 643 (s, υ_{TiO}), 605 (s) cm^{-1}. *Small amount of 1-hexanol due to adventitious decomposition of the complex in the presence of atmospheric moisture while collecting ATR-IR data in air.

Nuclear magnetic resonance (^{1}H NMR) data for [Ti(OCH$_2$CH$_2$CH$_2$CH$_2$CH$_2$CH$_3$)$_4$]

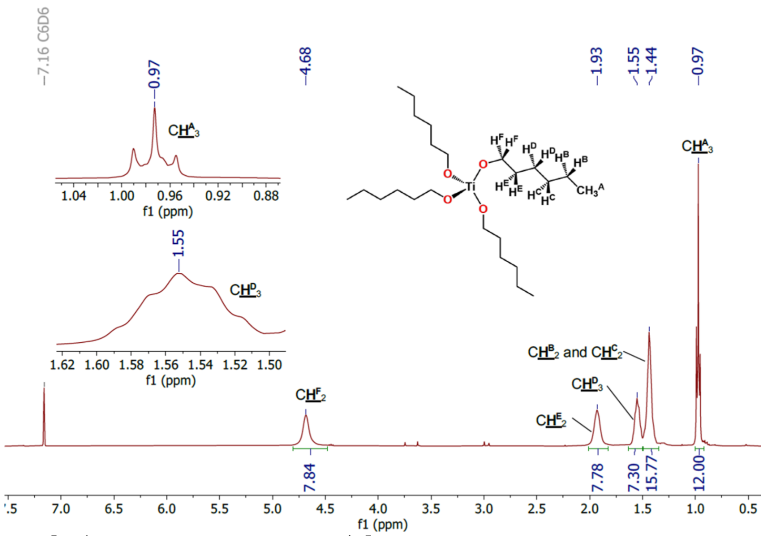

Figure S16. ^{1}H NMR spectrum of [Ti(OCH$_2$CH$_2$CH$_2$CH$_2$CH$_2$CH$_3$)$_4$], recorded in d_6-benzene.

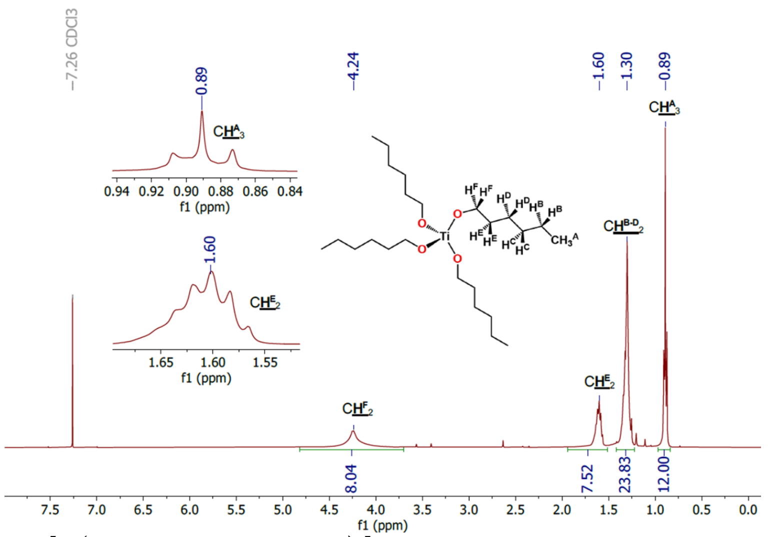

Figure S17. ^{1}H NMR spectrum of $[Ti(OCH_2CH_2CH_2CH_2CH_2CH_3)_4]$, recorded in d_3-chloroform.

Infrared (IR) data for $[Ti(OCH_2CH_2CH_2CH_2CH_2CH_3)_4]$

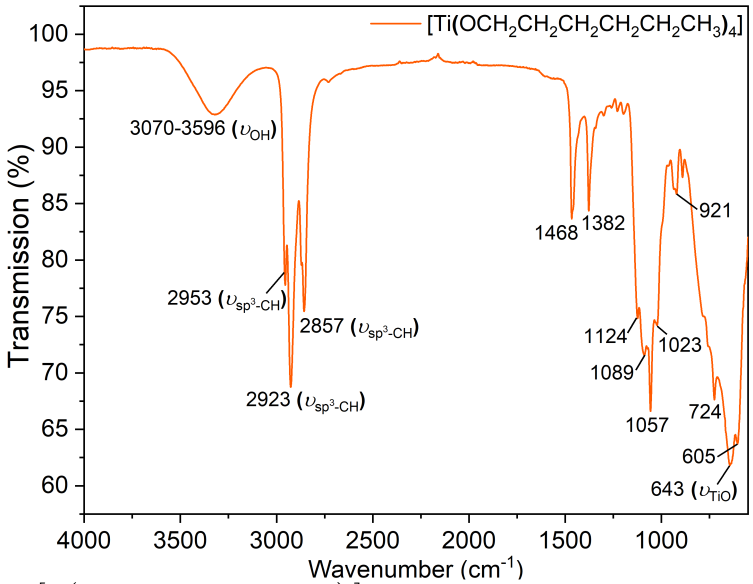

Figure S18. ATR-IR spectrum of $[Ti(OCH_2CH_2CH_2CH_2CH_2CH_3)_4]$.

REFERENCES

(1) Turton, D. A.; Senn, H. M.; Harwood, T.; Lapthorn, A. J.; Ellis, E. M.; Wynne, K. Terahertz Underdamped Vibrational Motion Governs Protein-Ligand Binding in Solution. *Nat. Commun.* **2014**, *5* (1), 3999. https://doi.org/10.1038/ncomms4999.

(2) González-Jiménez, M.; Barnard, T.; Russell, B. A.; Tukachev, N. V.; Javornik, U.; Hayes, L.-A.; Farrell, A. J.; Guinane, S.; Senn, H. M.; Smith, A. J.; Wilding, M.; Mali, G.; Nakano, M.; Miyazaki, Y.; McMillan, P.; Sosso, G. C.; Wynne, K. Understanding the Emergence of the Boson Peak in Molecular Glasses. *Nat. Commun.* **2023**, *14* (1), 215. https://doi.org/10.1038/s41467-023-35878-6.

(3) Neese, F. The ORCA Program System. *WIREs Comput. Mol. Sci.* **2012**, *2* (1), 73–78. https://doi.org/10.1002/wcms.81.

(4) Yanai, T.; Tew, D. P.; Handy, N. C. A New Hybrid Exchange-Correlation Functional Using the Coulomb-Attenuating Method (CAM-B3LYP). *Chem. Phys. Lett.* **2004**, *393* (1–3), 51–57. https://doi.org/10.1016/j.cplett.2004.06.011.

(5) Perdew, J. P.; Burke, K.; Ernzerhof, M. Generalized Gradient Approximation Made Simple. *Phys. Rev. Lett.* **1996**, *77* (18), 3865–3868. https://doi.org/10.1103/PhysRevLett.77.3865.

(6) Grimme, S.; Antony, J.; Ehrlich, S.; Krieg, H. A Consistent and Accurate Ab Initio Parametrization of Density Functional Dispersion Correction (DFT-D) for the 94 Elements H-Pu. *J. Chem. Phys.* **2010**, *132* (15), 154104. https://doi.org/10.1063/1.3382344.

(7) Grimme, S.; Ehrlich, S.; Goerigk, L. Effect of the Damping Function in Dispersion Corrected Density Functional Theory. *J. Comput. Chem.* **2011**, *32* (7), 1456–1465. https://doi.org/10.1002/jcc.21759.

(8) Weigend, F.; Ahlrichs, R. Balanced Basis Sets of Split Valence, Triple Zeta Valence and Quadruple Zeta Valence Quality for H to Rn: Design and Assessment of Accuracy. *Phys. Chem. Chem. Phys.* **2005**, *7* (18), 3297–3305. https://doi.org/10.1039/B508541A.

(9) Barone, V.; Cossi, M. Quantum Calculation of Molecular Energies and Energy Gradients in Solution by a Conductor Solvent Model. *J. Phys. Chem. A* **1998**, *102* (11), 1995–2001. https://doi.org/10.1021/jp9716997.
(10) Caughlan, C. N.; Smith, H. S.; Katz, W.; Hodgson, Wm.; Crowe, R. W. Organic Compounds of Titanium. II. Association of Organic Titanates in Benzene Solution. *J. Am. Chem. Soc.* **1951**, *73* (12), 5652–5654. https://doi.org/10.1021/ja01156a046.
(11) Babonneau, F.; Doeuff, S.; Leaustic, A.; Sanchez, C.; Cartier, C.; Verdaguer, M. XANES and EXAFS Study of Titanium Alkoxides. *Inorg. Chem.* **1988**, *27* (18), 3166–3172. https://doi.org/10.1021/ic00291a024.
(12) R.L. Martin; G. Winter. Structure of the Trinuclear Titanium (IV) Alkoxides. *Nature* **1960**, *188*, 313–315. https://doi.org/10.1038/188313a0.
(13) Ignatyev, I. S.; Montejo, M.; López González, J. J. DFT Predictions of Vibrational Spectra of Titanium Tetramethoxide Oligomers and the Structure of Titanium Tetraalkoxides in Liquid and Solid Phases. *Vib. Spectrosc.* **2009**, *51* (2), 218–225. https://doi.org/10.1016/j.vibspec.2009.05.001.
(14) Henkelman, G.; Uberuaga, B. P.; Jónsson, H. A Climbing Image Nudged Elastic Band Method for Finding Saddle Points and Minimum Energy Paths. *J. Chem. Phys.* **2000**, *113* (22), 9901–9904. https://doi.org/10.1063/1.1329672.
(15) Bitzek, E.; Koskinen, P.; Gähler, F.; Moseler, M.; Gumbsch, P. Structural Relaxation Made Simple. *Phys. Rev. Lett.* **2006**, *97* (17), 170201. https://doi.org/10.1103/PhysRevLett.97.170201.
(16) Trygubenko, S. A.; Wales, D. J. A Doubly Nudged Elastic Band Method for Finding Transition States. *J. Chem. Phys.* **2004**, *120* (5), 2082–2094. https://doi.org/10.1063/1.1636455.
(17) Sheppard, D.; Terrell, R.; Henkelman, G. Optimization Methods for Finding Minimum Energy Paths. *J. Chem. Phys.* **2008**, *128* (13), 134106. https://doi.org/10.1063/1.2841941.
